# Low-Protein Diets Differentially Regulate Energy Balance during Thermoneutral and Heat Stress in Cobb Broiler Chicken (*Gallus domesticus*)

**DOI:** 10.3390/ijms25084369

**Published:** 2024-04-15

**Authors:** Julia Sutton, Mohammad Habibi, Cedrick N. Shili, Ali Beker, Janeen L. Salak-Johnson, Andrew Foote, Adel Pezeshki

**Affiliations:** Department of Animal and Food Sciences, Oklahoma State University, Stillwater, OK 74078, USA; julia.sutton@okstate.edu (J.S.); habibi@wustl.edu (M.H.); cshili@alcorn.edu (C.N.S.); ali.beker@okstate.edu (A.B.); janeen.johnson@okstate.edu (J.L.S.-J.); andrew.foote@okstate.edu (A.F.)

**Keywords:** low-protein diet, thermoneutral, heat stress, energy balance

## Abstract

The objective was to assess whether low-protein (LP) diets regulate food intake (FI) and thermogenesis differently during thermoneutral (TN) and heat stress (HS) conditions. Two-hundred-day-old male broiler chicks were weight-matched and assigned to 36 pens with 5–6 chicks/pen. After 2 weeks of acclimation, birds were subjected into four groups (9 pens/group) including (1) a normal-protein diet under TN (ambient temperature), (2) an LP diet under TN, (3) a normal-protein diet under HS (35 °C for 7 h/day), and (4) an LP diet under HS, for 4 weeks. During HS, but not TN, LP tended to decrease FI, which might be associated with a lower mRNA abundance of duodenal ghrelin and higher GIP during HS. The LP group had a higher thermal radiation than NP under TN, but during HS, the LP group had a lower thermal radiation than NP. This was linked with higher a transcript of muscle β1AR and AMPKα1 during TN, but not HS. Further, LP increased the gene expression of COX IV during TN but reduced COX IV and the sirtuin 1 abundance during HS. The dietary protein content differentially impacted plasma metabolome during TN and HS with divergent changes in amino acids such as tyrosine and tryptophan. Compared to NP, LP had increased abundances of *p_Tenericutes*, *c_Mollicutes*, *c_Mollicutes_RF9*, and *f_tachnospiraceae* under HS. Overall, LP diets may mitigate the negative outcome of heat stress on the survivability of birds by reducing FI and heat production. The differential effect of an LP diet on energy balance during TN and HS is likely regulated by gut and skeletal muscle and alterations in plasma metabolites and cecal microbiota.

## 1. Introduction

During heat stress (HS), a homeostatic effort enhances the survivability of birds by reducing the body temperature via decreasing food intake (FI) and thermogenesis [1,2]. A low-protein (LP) diet balanced with limiting essential amino acids has been suggested as a promising dietary solution to reduce the mortality of birds under HS conditions [3,4,5]. Compared to carbohydrates and lipids, proteins have a high heat increment; hence, offering LP diets may decrease metabolic heat production [1,5,6]. LP diets have been shown to reduce FI, heat production, and mortality of broilers under HS [3,7,8]; however, high-protein diets fed to broilers under HS conditions increase heat production [5]. Reduced FI in response to LP diets during HS also decreases thermogenesis and body core temperature [8]. Unlike HS conditions, although not consistently, LP-induced hyperphagia [9,10,11] and thermogenesis [10,12,13,14,15] have been reported in birds under thermoneutral (TN) conditions. The hyperphagic response to LP diets in chickens has been explained as an effort to meet the protein needs, which can instead result in increased energy expenditure [13]. Therefore, independent studies provide evidence that LP diets induce differential effects on energy balance under TN or HS; however, this has not been tested under a single experiment. Further, less is understood about the pathways by which LP diets regulate FI and thermogenesis during TN and HS conditions.

Changes in FI in birds fed with LP diets have been previously related to amino acid imbalances and the rate of their absorption and net energy/metabolic energy ratio [16]. The role of gut peptides in the regulation of FI in poultry is well known [17,18,19]. Dietary protein likely induces glucagon-like peptide-1 (GLP-1) secretion from ileal L cells in birds via essential amino acids such as lysine and methionine [20,21]. Therefore, an LP-induced increase in FI is possibly due to the reduced secretion of anorexigenic gut peptides like GLP-1. In one study, feeding broilers with LP diets under TN reduced the immunoreactive GLP-1 cells in the ileum [20]. The effect of LP diets on the gene expression of gut peptides during HS is not known. The relative importance of gut peptides in the differential effects of LP diets on FI under TN and HS is little understood.

Changes in the thermogenesis of birds fed with LP diets have been associated with alterations in the concentration of hormones such as triiodothyronine [13] and amino acids [16]. Skeletal muscle plays a key role in the regulation of thermogenesis in birds [17]. LP diets have been shown to increase the expression of Avian uncoupling protein (avUCP) and adenine nucleotide translocase in the skeletal muscles of boilers under TN conditions [11,15]. These studies suggest that LP-induced thermogenesis is controlled by avUCP in birds under TN. The effect of LP diets on muscle thermogenesis during HS is overlooked. It is unknown whether the divergent effect of LP diets on energy expenditure during TN and HS conditions is mediated by factors controlling muscle thermogenesis.

Environmental stressors such as heat stress can modulate the composition of gut microbiota and the profile of blood metabolites in birds [22,23,24,25]. Under TN conditions, the LP diet increased the population of cecal *Lactobacillaceae* [26] and the concentration of blood metabolites such as amino acids, α-aminoadipic acid, and serotonin [27]. Changes in gut microbiota composition and blood metabolites due to dietary alterations may influence the energy balance and adaptability to heat stress. The gut microbiome and blood metabolomics profile of birds fed with LP diets under HS have not been fully characterized. Little is understood about whether the differential effects of LP diets on energy balance under TN and HS are associated with alterations in the population of gut bacteria and plasma metabolites. The objective of this study was to assess the effects of LP diets on energy balance, body composition, cecal microbiota composition, and plasma metabolomics in broilers under experimentally induced HS.

## 2. Results

### 2.1. Survival

**Figure 1:**
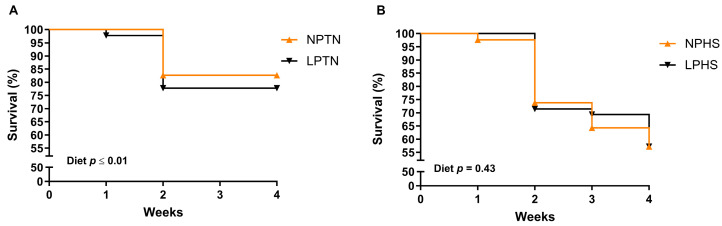
Survival curve of broilers fed with low-protein diets during experimentally induced heat stress. Effect of low-protein diets on survival (%) of broilers during thermoneutral (TN) (**A**) and heat stress (HS) (**B**). NPTN: normal-protein diet under TN; LPTN: low-protein diet under TN; NPHS: normal-protein diet under HS; LPHS: low-protein diet under HS. The *p*-values for the overall model effects for diet, temperature (temp), week, diet × temp, diet × week, temp × week, and diet × week × temp were 0.27, ≤0.01, ≤0.01, 0.02, 0.14, ≤0.01, 0.38. *n* = 9 per treatment.

Overall, the effect of temperature on the survival of birds was significant with birds in the HS group, having a lower survival (%) than those assigned to TN (67.4% vs. 69.8%, respectively). Further, the effect of diet × temperature on the survival of birds was significant. This was due to the significant diet effect on the survival of birds within the TN group but not in the HS (Figure 1A,B). Although low-protein diet under TN (LPTN) had a lower survival rate than normal-protein diet under TN (NPTN; 77.8% vs. 82.6%, respectively), there was no difference in the survival rate between the low-protein diet under HS (LPHS) and normal-protein diet under HS (NPHS; 57.1% vs. 57.1%) groups.

### 2.2. Behavioral Adaptations

The effect of temperature on eating, panting, wing spread, respiratory rate, and rectal temperature was significant (Table 1). The eating was decreased (3.3 vs. 9.6%, respectively), and panting, wing spread, respiratory rate, and rectal temperature were increased during HS (86.9 vs. 2.7%, 27.9 vs. 0%, 199.7 vs. 66.1 breath/min, and 42.1 vs. 40.4 °C, respectively). The effect of diet on drinking was significant, with LP having lower drinking (%) than NP (6.1 and 8.1%, respectively). Compared to NPTN, LPTN had reduced drinking by ~32%, increased panting by ~94%, and tended to increase the respiratory rate by ~3%. Compared to NPHS, LPHS had reduced drinking by ~16%.

When the data were analyzed for each phase (i.e., grower and finisher phase; Figure S1A–L), LPTN increased the respiratory rate and panting (%) in the grower phase compared to NPTN (~3.8% and 85.3%, respectively). Compared to NPTN, LPTN increased eating (%) and panting (%) in the finisher phase (~25.1% and 103.4%, respectively). Compared to NPHS, LPHS decreased drinking (%) in the grower phase (~32.1%). Relative to NPHS, LPHS decreased the rectal temperature, the respiratory rate, and drinking (%) in the finisher phase (~1.0%, 0.4% and 24.2%, respectively).

### 2.3. Growth Measurements

The initial body weight (BW) and the final BW were not different among dietary groups (Table 2). The effect of diet on the average daily gain (ADG), average daily protein intake (ADPI), average daily water intake (ADWI), and water: food (W:F) ratio was significant, with lower values for all of them for LP compared to NP (59 vs. 66 g/d, 24 vs. 30 g/d, 269 vs. 346 mL/d, 1.8 vs. 2.1 g/g for LP vs. NP). The effect of temperature on the ADG, average daily food intake (ADFI), ADPI, ADWI, gain: protein (G:P) ratio, and W:F ratio was significant, with lower values for all of them for HS compared to TN, except ADWI, the G:P ratio, and the W:F ratio (49 vs. 76 g/d, 149 vs. 175 g/d, 21 vs. 31 g/d, 335 vs. 280 mL/d, 4.2 vs. 2.8 g/g, and 2.3 vs. 1.6 mL/g, respectively). The effect of diet × temperature on ADFI, ADPI, G:P, and W:F was significant. Although no differences in the ADFI were detected between the NPTN and LPTN groups, LPHS tended to reduce the ADFI compared to NPHS. Compared to NPTN, LPTN reduced the ADPI, ADWI, gain: food (G:F) ratio, and W:F ratio but increased the G:P ratio (~25%, 24%, 20%, 32%, and 20%, respectively). Compared to NPHS, LPHS decreased the ADG and ADWI (~19% and 20%) and tended to decrease the ADFI and ADPI (~11% and 17%, respectively).

When the data were analyzed separately for each phase (grower and finisher), LPTN reduced the ADPI, ADWI, and W:F ratio but increased the G:P ratio compared to NPTN in the grower phase (~22%, 20%, 22%, and 32%, respectively; Table S1). Relative to NPHS, LPHS decreased the ADG, ADFI, ADPI, and ADWI but increased the G:P ratio in the grower phase (~31%, 19%, 41%, 13%, and 25%, respectively). In the finisher phase, compared to NPTN, LPTN reduced the ADPI, ADWI, and W:F ratio and tended to reduce the G:F ratio but increased the G:P ratio (~24%, 27%, 30%, 9%, and 23%, respectively). Relative to NPHS, LPHS tended to decrease the ADG and decreased the ADPI, ADWI, and W:F ratio but increased the G:P ratio (~12%, 33%, 22%, 15%, and 28%, respectively).

### 2.4. Body Composition Analysis

The effect of diet on lean mass, lean percent, fat mass, and fat percent were significant (Table 3). Compared to NP, LP-fed birds had a lower lean mass and lean percent and had a higher fat mass and fat percent (2252.2 vs. 2505.2 g, 90.1 vs. 94.3%, 212.5 vs. 122.2 g, 8.3 vs. 4.5%, respectively). The effect of temperature on the bone mineral content (BMC), BMC percent, bone mineral density (BMD), lean mass, lean percent, fat mass, and fat percent was significant (Table 3). Compared to TN, birds under HS had a lower BMC, BMC percent, BMD, lean mass, fat mass, and fat percent (25.1 vs. 34.8 g, 1.1 vs. 1.2%, 0.14 vs. 0.15 g/cm^2^, 2123.0 vs. 2637.4 g, 126.08 vs. 208.7 g, 5.6 vs. 7.2%, respectively). The BMC, BMC percent, and BMD were not different among dietary groups (Table 3). Compared to NPTN, LPTN reduced the lean mass and lean percent but increased the fat mass and fat percent (~8%, 7%, 110%, and 123%, respectively). Compared to NPHS, LPHS decreased the lean mass and lean percent but increased the fat mass and fat percent (~12%, 2%, 30%, and 45%, respectively). The effect of diet × temperature on the lean percent, fat mass, and fat percent was significant, which was due to the more significant effect of diet on changing these parameters under TN than HS.

### 2.5. Radiative Heat Transfer

**Figure 2:**
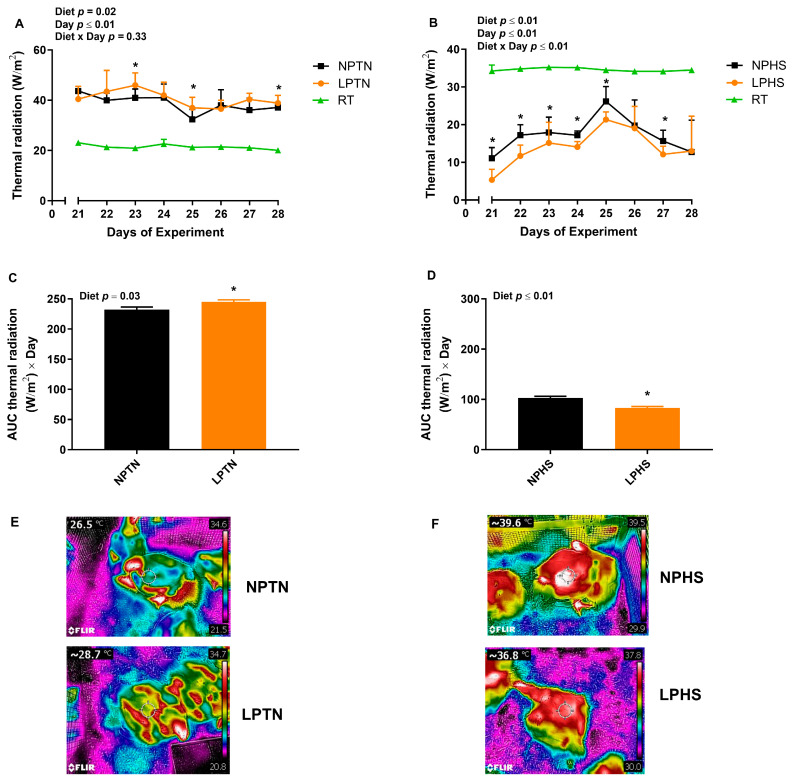
Thermal radiation of broilers fed with low-protein diets during experimentally induced heat stress. Effect of low-protein diets on thermal radiation (**A**,**B**) and the area under the curve (AUC) of thermal radiation (**C**,**D**) of broilers during thermoneutral (TN) (**A**,**C**) and heat stress (HS) (**B**,**D**). A representative screenshot of a thermal image for broilers during TN (**E**) and HS (**F**). NPTN: normal-protein diet under TN; LPTN: low-protein diet under TN; NPHS: normal-protein diet under HS; LPHS: low-protein diet under HS. RT: room temperature. The *p*-values for the overall model effects of diet, temperature (temp), day, diet × temp, diet × day, day × temp, and diet × temp × day for thermal radiation were 0.99, ≤0.01, ≤0.01, ≤0.01, 0.56, ≤0.01, 0.03, respectively. The *p*-values for the overall model effects for diet, temp, and diet × temp for AUC thermal radiation were 0.75, ≤0.01, and ≤0.01, respectively. Asterisks (*) in the bar plots indicate a significant difference (*p* ≤ 0.05, *t*-test). The values are the mean ± standard errors of means; *n* = 9.

The overall effect of temperature and diet × temperature on the thermal radiation was significant (Figure 2). Birds under HS had lower thermal radiation than those kept under TN (14.6 vs. 39.1 W/m^2^, respectively). While LPTN had higher thermal radiation than NPTN (40.6 vs. 37.5 W/m^2^, respectively), LPHS had lower thermal radiation than NPHS (13.0 vs. 16.1 W/m^2^, respectively) (Figure 2A,B). LPTN had higher thermal radiation on days 23, 25, and 27 (~30%, 14%, and 6%, respectively; Figure 2A). Compared to NPHS (Figure 2B), LPHS had lower thermal radiation on days 21, 22, 23, 24, 25, and 27 (~52%, 32%, 15%, 18%, 21%, and 23%, respectively).

When thermal radiation was expressed as the area under the curve (AUC), the effect of temperature and diet × temperature on the thermal radiation AUC was significant. The HS group had a lower thermal radiation AUC than the TN group (92.8 vs. 236.5 (W/m^2^) × day, respectively). Compared to NPTN, LPTN increased the thermal radiation AUC by ~8% (Figure 2C), but relative to NPHS, LPHS decreased the thermal radiation AUC by ~19% (Figure 2D).

### 2.6. Reverse Transcription Polymerase Chain Reaction (RT-qPCR)

**Figure 3:**
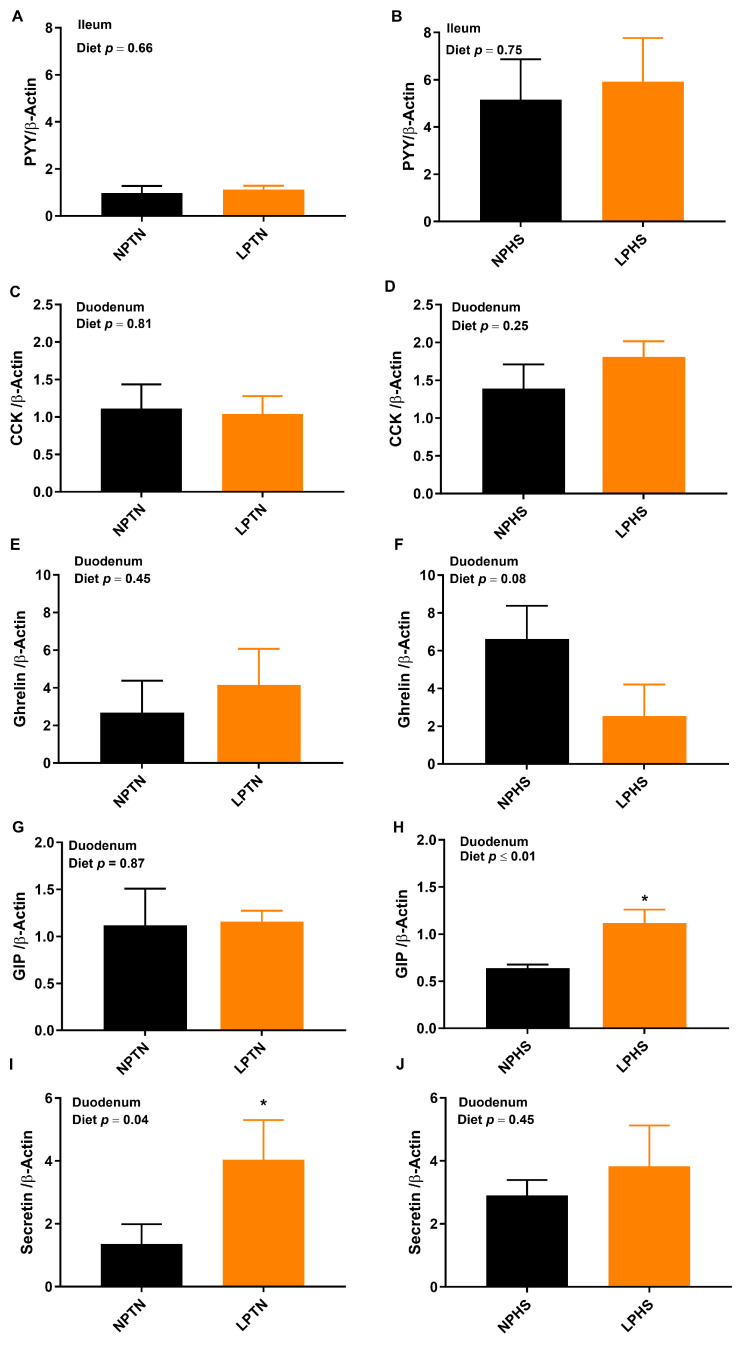
mRNA abundance of food intake markers in the duodenum or ileum of broilers fed low-protein diets during experimentally induced heat stress. Effect of low-protein diets on mRNA abundance of peptide YY (PYY) (**A**,**B**), cholecystokinin (CCK) (**C**,**D**), ghrelin (**E**,**F**), gastric inhibitory polypeptide (GIP) (**G**,**H**), and secretin (**I**,**J)** in duodenum or ileum of broilers during thermoneutral (TN) (**A**,**C**,**E**,**G**,**I**) and heat stress (HS) (**B**,**D**,**F**,**H**,**J**). NPTN: normal-protein diet under TN; LPTN: low-protein diet under TN; NPHS: normal-protein diet under HS; LPHS: low-protein diet under HS. The *p*-values for the overall model effects of diet, temperature (temp), and diet × temp for PYY were 0.74, ≤0.01, 0.82, for CCK were 0.45, 0.03, 0.29, for ghrelin were 0.37, 0.42, 0.07, for GIP were 0.05, 0.05, 0.10, and for secretin were 0.04, 0.42, 0.29. Asterisks (*) in the bar plots indicate a significant difference (*p* ≤ 0.05, *t*-test). The values are the mean ± standard errors of means; *n* = 8.

The overall diet effect on the mRNA abundance of gastric inhibitory polypeptide (GIP) and secretin in the duodenum was significant, with the LP group having higher values than NP (Figure 3G–J). The overall temperature effect on the mRNA abundance of peptide YY (PYY) in the ileum and cholecystokinin (CCK) in the duodenum was significant, with HS having a higher mRNA abundance of PYY and CCK than TN (Figure 3A–D). The effect of diet × temperature on the mRNA abundance of ghrelin in duodenum and GIP in duodenum tended to be significant. Although no differences were detected between the mRNA abundance of ghrelin in NPTN vs. LPTN, LPHS tended to decrease it by ~62% in comparison with NPHS (Figure 3E,F). No differences in the abundance of GIP were detected between the NPTN and LPTN groups, but LPHS had a higher (~75%) GIP transcript than NPHS (Figure 3G,H). No differences in the transcript abundance of ileum PYY and duodenum CCK and ghrelin were detected when both NPTN vs. LPTN and NPHS vs. LPHS were compared (Figure 3A–F).

**Figure 4:**
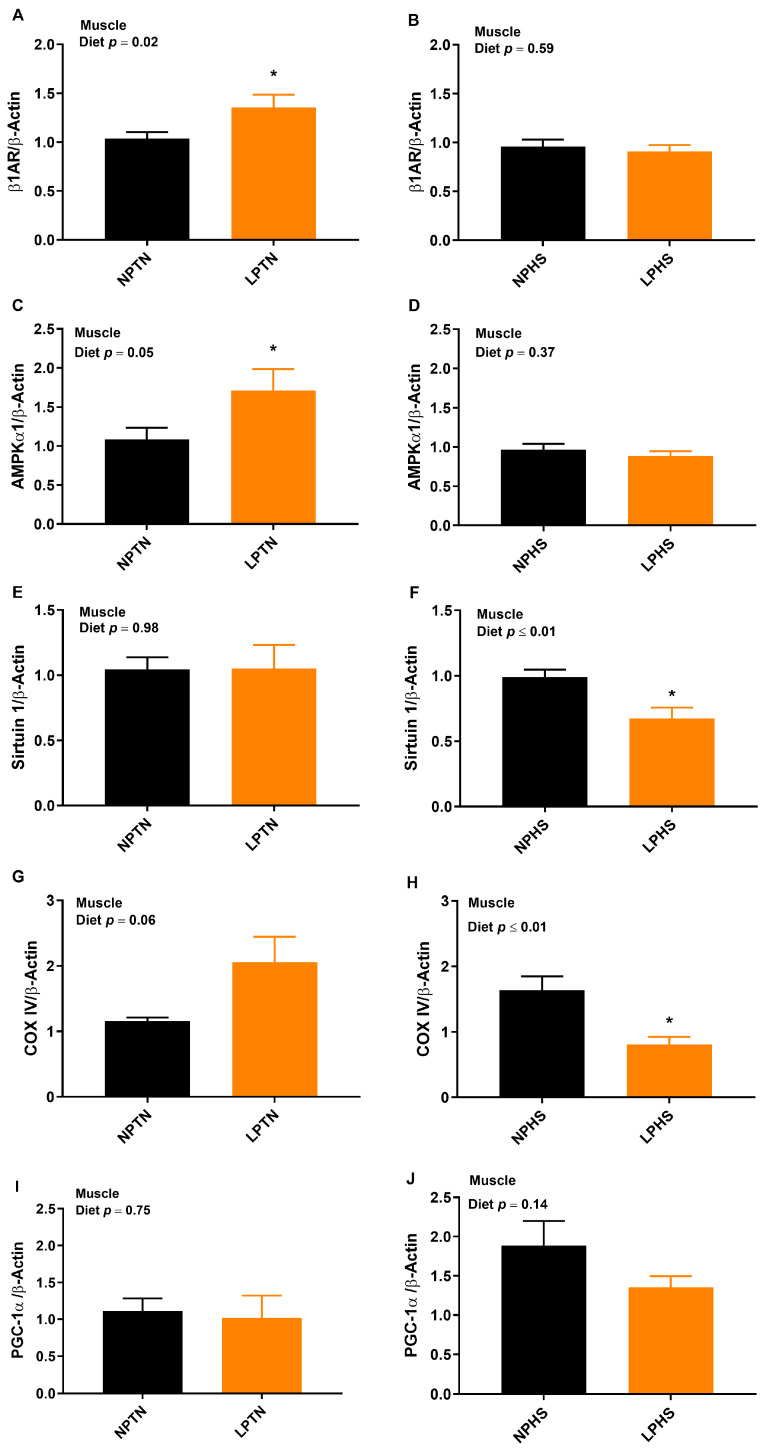
mRNA abundance of thermogenesis markers in muscle of broilers fed low-protein diets during experimentally induced heat stress. Effect of low-protein diets on mRNA abundance of β-1 adrenergic receptor (β1AR) (**A**,**B**), AMP-activated protein kinase α1 (AMPKα1) (**C**,**D**), sirtuin 1 (**E**,**F**), cytochrome c oxidase subunit IV (COX IV) (**G**,**H**), and peroxisome proliferator-activated receptor- gamma coactivator 1α (PGC-1α) (**I**,**J**) in the muscle in broilers during thermoneutral (TN) (**A**,**C**,**E**,**G**,**I**) and heat stress (HS) (**B**,**D**,**F**,**H**,**J**). NPTN: normal-protein diet under TN; LPTN: low-protein diet under TN; NPHS: normal-protein diet under HS; LPHS: low-protein diet under HS. The *p*-values for the overall model effects of diet, temperature (temp), and diet × temp for β1AR were 0.08, ≤0.01, 0.02, for AMPKα1 were 0.07, ≤0.01, 0.02, for sirtuin 1 were 0.07, 0.02, 0.06, for COX IV were 0.88, 0.12, ≤0.01, and for PGC-1α were 0.17, 0.02, 0.32. Asterisks (*) in the bar plots indicate a significant difference (*p* ≤ 0.05, *t*-test). The values are the mean ± standard errors of means; *n* = 8.

The overall diet effect on the mRNA abundance of muscle β-1 adrenergic receptor (β1AR), AMP-activated protein kinase α1 (AMPKα1), and sirtuin 1 tended to be significant with the LP group having higher values for β1AR and AMPKα1 and lower values for sirtuin 1 compared to NP (Figure 4A–F). The overall temperature effect on the mRNA abundance of muscle β1AR, AMPKα1, sirtuin 1, and peroxisome proliferator-activated receptor- gamma coactivator 1α (PGC-1α) was significant, with HS having lower values for β1AR, AMPKα1, and sirtuin 1 but a higher value for PGC-1α than TN (Figure 4A–F,I,J). The overall effect of diet × temperature for muscle β1AR, AMPKα1, and cytochrome c oxidase subunit IV (COX IV) (Figure 4A–D,G,H) was significant. Compared to NPTN, LPTN increased muscle β1AR and AMPKα1 transcript (~30% and 58%, respectively), but no differences were detected when NPHS and LPHS were compared. Although LPTN tended to increase the mRNA abundance of muscle COX IV in comparison with NPTN, relative to NPHS, it was decreased in LPHS by ~50%. The overall effect of diet × temperature for muscle sirtuin 1 (Figure 4E,F) was significant. While no differences were detected in the muscle sirtuin 1 transcript between NPTN and LPTN, relative to NPHS, it was decreased in LPHS by ~32%. No difference in the transcript abundance of muscle PGC-1α was detected when NPTN vs. LPTN or NPHS vs. LPHS were compared (Figure 4I,J).

### 2.7. Oxidative Stress Biomarkers in Plasma

**Figure 5:**
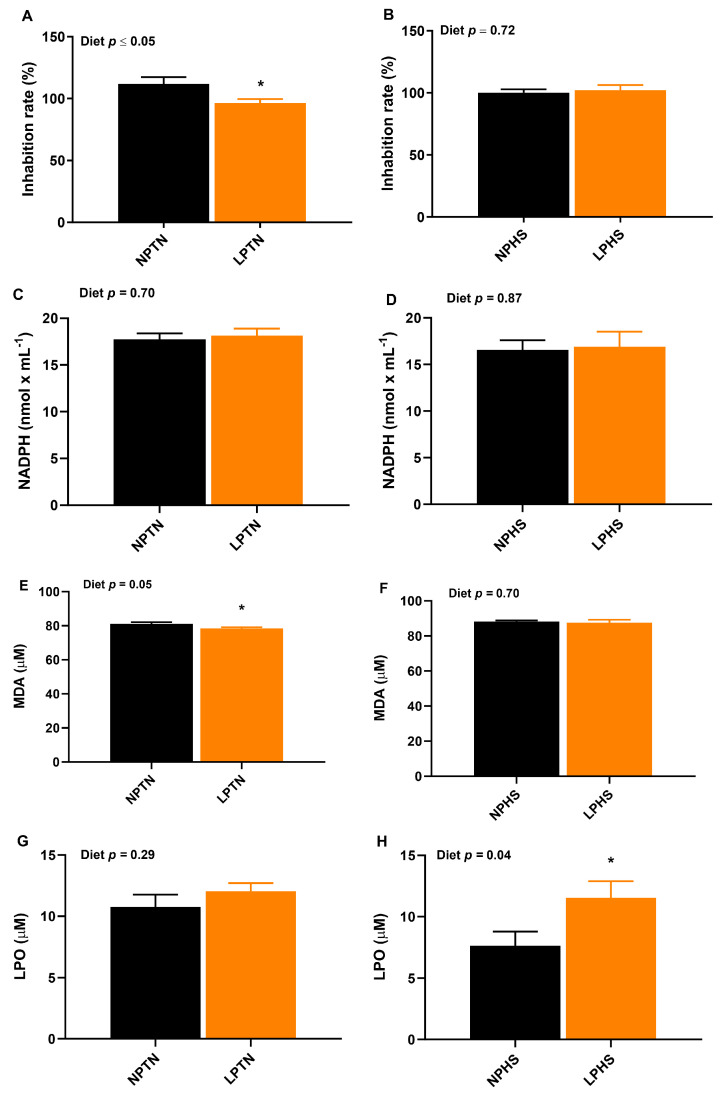
Superoxide dismutase activity, glutathione peroxidase activity, and lipid peroxidation in plasma of broilers fed with low-protein diets during experimentally induced heat stress. Effect of low-protein diets on superoxide dismutase (SOD) activity (**A**,**B**), glutathione peroxidase (GPx) activity (**C**,**D**), lipid peroxidation of malondialdehyde (MDA) (**E**,**F**), and lipid hydroperoxide (LPO) (**G**,**H**) of broilers during thermoneutral (TN) (A,C,E) and heat stress (HS) (**B**,**D**,**F**). NPTN: normal-protein diet under TN; LPTN: low-protein diet under TN; NPHS: normal-protein diet under HS; LPHS: low-protein diet under HS. The *p*-values for the overall model effects for diet, temperature (temp), diet × temp, for SOD activity were 0.12, 0.49, 0.05, for GPx activity were 0.74, 0.28, 0.98, for MDA were 0.12, ≤0.01, 0.35, and for LPO were 0.02, 0.11, and 0.24. Asterisks (*) in the bar plots indicate a significant difference (*p* ≤ 0.05, *t*-test). The values are the mean ± standard errors of means. *n* = 5–6 per treatment for SOD activity, *n* = 9 per treatment for GPx activity, MDA and LPO.

The effect of diet × temperature on superoxide dismutase (SOD) activity was significant. This was due to the significant diet effect on SOD activity within the TN group but not in the HS group (Figure 5A,B). Compared to NPTN, LPTN reduced SOD activity by ~14%. The effect of temperature on plasma malondialdehyde (MDA) was significant, with HS having higher values for MDA than TN (88.0 vs. 79.8 μM, respectively). Relative to NPTN, LPTN reduced MDA concentrations by ~3% (Figure 5E). The overall diet effect on lipid hydroperoxide (LPO) was significant, with LP having a higher value for LPO compared to NP (11.8 vs. 9.2 μM, respectively). Compared to NPHS, LPHS increased the concentration of LPO by 51% (Figure 5H).

### 2.8. Plasma Metabolites

**Figure 6:**
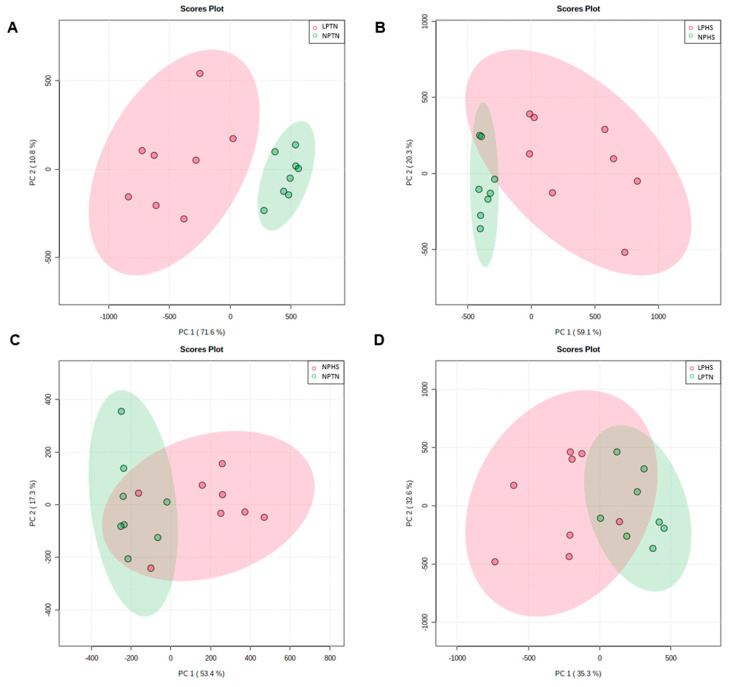
Principal component analysis (PCA) score plots of plasma metabolites in broilers fed with low-protein diets during experimentally induced heat stress. PCA score plots of plasma metabolites for NPTN vs. LPTN (**A**), NPHS vs. LPHS (**B**), NPTN vs. NPHS (**C**), and LPTN vs. LPHS (**D**). NPTN: normal-protein diet under TN; LPTN: low-protein diet under TN; NPHS: normal-protein diet under HS; LPHS: low-protein diet under HS. Each node represents an individual bird. *n* = 8.

The principal component analysis (PCA) score plot displays a clear separation between NPTN and LPTN for plasma metabolites (Figure 6A). There was a positive loading for NPTN and a negative loading for LPTN on the PC1 axis. PC1 explains 71.6% of the variation in the metabolite changes within the samples, and PC2 is indicative of 10.8% of the variation. The PCA score plot between NPHS and LPHS (Figure 6B) displays a separation between these two groups for plasma metabolites. There was a negative loading for NPHS and a positive loading for LPHS on the PC1 axis. PC1 explains 59.1% of the variation in the metabolite changes within the samples, and PC2 is indicative of 20.3% of the variation. The PCA score plot between NPHS and NPTN (Figure 6C) displays a slight separation between the two groups. There was primarily a positive loading for NPHS and a negative loading for NPTN on the PC1 axis. PC1 explains 53.4% of the variation in the metabolite changes within the samples, and PC2 is indicative of 17.3% of the variation. The PCA score plot comparing LPHS and LPTN (Figure 6D) displays a slight separation between the two groups. There was a negative loading for LPHS and a positive loading for LPTN on the PC2 axis. PC1 explains 35.3% of the variation in the metabolite changes within the samples, and PC2 is indicative of 32.6% of the variation.

**Figure 7:**
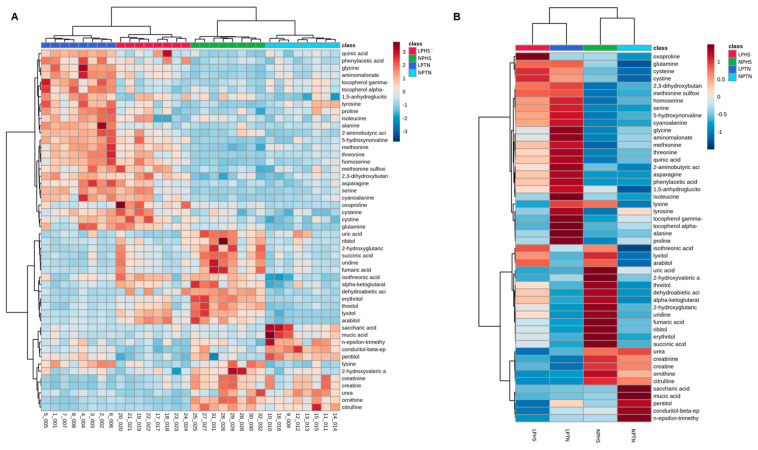
Heat map of plasma metabolites in broilers fed with low-protein diets during experimentally induced heat stress. Hierarchical clustering of all significantly different plasma metabolites in birds used in the current study (**A**) and among birds that received NPTN, LPTN, LPHS, and HPHS (**B**). The rows display metabolites, and the columns represent the birds (Figure 4A) or experimental groups (Figure 4B). The dark red or blue box corresponds to the magnitude of difference when compared with the average value. The black dendrogram along the left side of the heatmap indicates both the similarity and the order of the clusters that were formed. NPTN: normal-protein diet under TN; LPTN: low-protein diet under TN; NPHS: normal-protein diet under HS; LPHS: low-protein diet under HS. *n* = 8.

**Figure 8:**
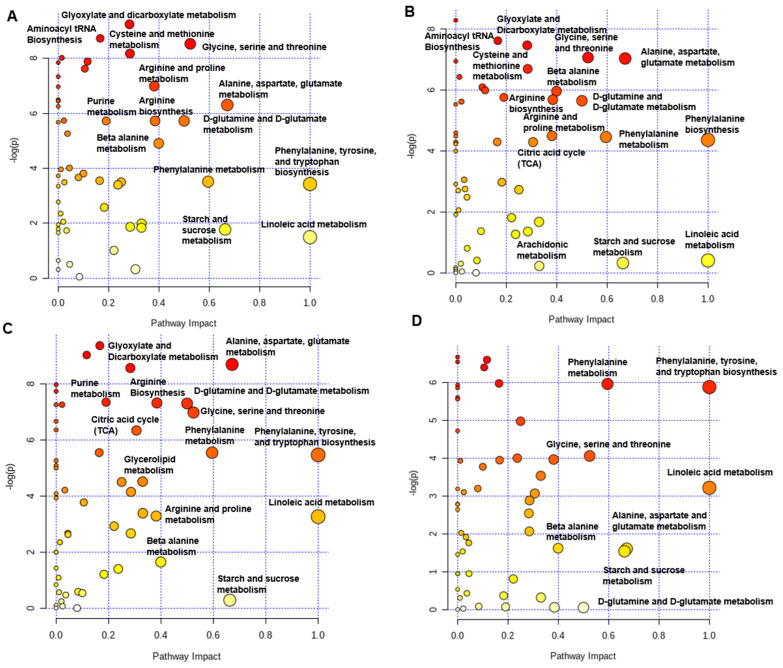
Pathway analysis map of plasma metabolites in broilers fed with low-protein diets during experimentally induced heat stress. The map of the pathway analysis for the metabolites detected in the blood serum of broilers fed with NPTN vs. LPTN (**A**), NPHS vs. LPHS (**B**), NPTN vs. NPHS (**C**), and LPTN vs. LPHS (**D**). Each circle shows a metabolic pathway. The scores were obtained from topology analysis with pathway impact (x axis) and the pathway enrichment analysis (y axis). The color of each circle is a function of its *p*-value and pathway enrichment, while the size of each circle is determined on the basis of its impact value. Therefore, the darker color circles show the metabolites with more significant changes and higher pathway enrichment, and the larger circles are the ones with higher pathway impact. NPTN: normal-protein diet under thermoneutral; LPTN: low-protein diet under thermoneutral; NPHS: normal-protein diet under heat stress; LPHS: low-protein diet under heat stress. *n* = 8.

The hierarchical clustering heat map of significantly different plasma metabolites detected differentially expressed metabolites in plasma when the data were analyzed for individual birds (Figure 7A) or as a treatment group (Figure 7B). The metabolic pathway enrichment analysis showed that, when comparing NPTN and LPTN, the aminoacyl tRNA biosynthesis, glyoxylate and dicarboxylate metabolism, glycine, serine and threonine metabolism, alanine, aspartic acid and glutamate metabolism, phenylalanine, tyrosine, and tryptophan biosynthesis, and linoleic acid metabolism pathways were influenced (Figure 8A). When NPHS was compared to LPHS, the aminoacyl tRNA biosynthesis, glyoxylate and dicarboxylate metabolism, glycine, serine and threonine metabolism, alanine, aspartic acid and glutamate metabolism, phenylalanine metabolism, phenylalanine biosynthesis, and linoleic acid metabolism pathways were changed (Figure 8B). Comparing NPTN with NPHS, the glyoxylate and dicarboxylate metabolism, alanine, aspartic acid, and glutamate metabolism, D-glutamine and D-glutamate metabolism, glycine, serine, and threonine metabolism, phenylalanine, tyrosine, and tryptophan biosynthesis, starch and sucrose metabolism, and linoleic acid metabolism were affected (Figure 8C). When LPTN was compared with LPHS, the phenylalanine metabolism, phenylalanine, tyrosine, and tryptophan biosynthesis, linoleic acid metabolism, alanine, aspartate, and glutamate metabolism, starch and sucrose metabolism, and D-glutamine and D-glutamate metabolism were changed (Figure 8D). Overall, metabolic pathway enrichment analysis showed that metabolites involved in the carbohydrate, lipid, protein, and amino acid metabolism were affected with experimental treatments.

All the plasma metabolites that changed significantly among dietary groups are given in Table 4, while the non-significant plasma metabolites are presented in Table S2. Experimental groups affected the plasma metabolites related with amino acids and amino acid derivatives. Compared to NPTN, LPTN reduced aspartic acid, valine, tryptophan, and tyrosine but increased lysine, asparagine, isoleucine, alanine, cysteine, cysteine, glutamine, glycine, methionine, proline, serine, and threonine. Compared to NPHS, LPHS tended to reduce aspartic acid, leucine, threonine, homocystine, and histidine but increased tyrosine, asparagine, cysteine, glutamine, glycine, methionine, and serine. For protein metabolism, compared to NPTN, LPTN decreased creatinine, ornithine, urea, and citrulline. Compared to NPHS, LPHS decreased creatinine, ornithine, urea, uric acid, and citrulline. For the amino acid metabolism, relative to NPTN, LPTN increased 3-hydroxyanthralinic acid and aminomalonate. Compared to NPHS, LPHS decreased fumaric acid and tended to decrease ketoisocaproic acid but increased aminomalonate and guanidinosuccinate.

Compared to NPTN, LPTN reduced dihydroxypyrazine, pipecolinic acid, and serotonin and tended to decrease maleimide, phosphoethanolamine, and pimelic acid but increased 5-hydroxynorvaline, spermidine, and trans-4-hydroxyproline. Compared to NPHS, LPHS decreased cytosine and tended to decrease malic acid but tended to increase dihydroxypyrazine and increased 5-hydroxynorvaline, maleimide, phosphoethanolamine, pimelic acid, pipecolinic acid, spermidine, and trans-4-hydroxyproline.

For carbohydrates, compared to NPTN, LPTN decreased fructose-6-phosphate, glucose-6-phosphate, raffinose, xylose, and tended to decrease ribose, maltose, and sucrose but increased anhydroglucitol and arabitol. When compared to NPHS, LPTN decreased ribose, erythritol, erythrose, galactitol, glucose-1-phosphate, ribitol, sorbital, tagatose, and threitol and tended to decrease alpha-ketoglutarate. For carbohydrate derivatives, relative to NPTN, LPTN reduced azelaic acid, conduritol-beta-epoxide, quinic acid, mucic acid, pinitol, and saccharic acid and tended to decrease melezitose, ribonic acid, and xylitol. Compared to NPHS, LPHS decreased conduritol-beta-epoxide, gluconic acid, pinitol, quinic acid, and uridine and tended to decrease xylitol but increased azelaic acid and tended to increase inulotriose.

For the fatty acid metabolism, relative to NPTN, LPTN decreased 2-deoxytetronic acid, glycerol-alpha-phosphate, lauric acid, and palmitoleic acid and tended to decrease cholesterol but increased 2-aminobutyric acid. Compared to NPHS, LPHS decreased 2-hydroxyglutaric acid, 2-hydroxyvaleric acid, 3-hydroxybutyric acid, and glycerol-alpha-phosphate but tended to increase 2-aminobutyric acid and cholesterol.

### 2.9. Cecal Microbiota

The rarefaction curve analysis showed that all samples analyzed reached a stable plateau at 40,000 reads for each sample, suggesting that the sequencing depth was sufficient to saturate the bacterial communities in cecal samples (Figure S2).

**Figure 9:**
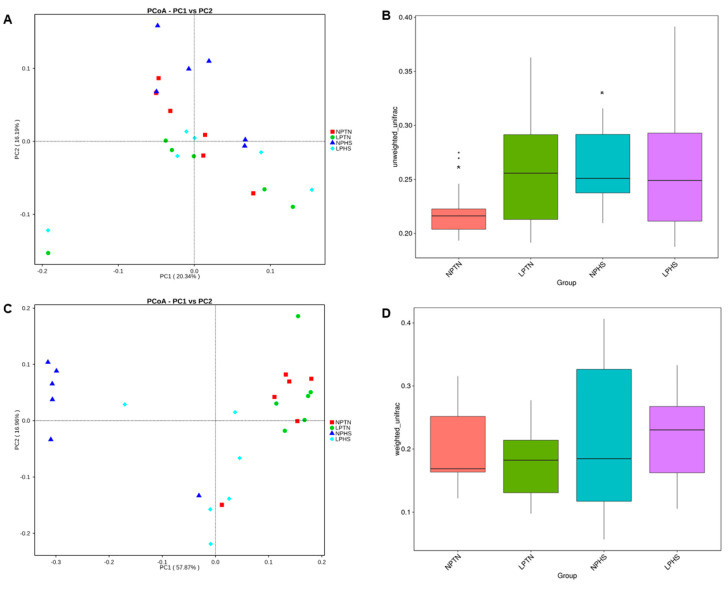
Beta diversity of cecal bacterial community in broilers fed with low-protein diets during experimentally induced heat stress. Principal coordinate analysis (PCoA) of unweighted UniFrac distances, representing the diversity of cecal bacterial populations across all birds assigned to 4 treatments with each node being indicative of an individual bird (**A**), unweighted UniFrac distances shown across dietary groups (**B**), PCoA of weighted UniFrac distances, representing the diversity of cecal bacterial populations across all birds assigned to 4 treatments, with each node being indicative of an individual bird (**C**), and weighted UniFrac distances shown across dietary groups (**D**). NPTN: normal-protein diet under thermoneutral; LPTN: low-protein diet under thermoneutral; NPHS: normal-protein diet under heat stress; LPHS: low-protein diet under heat stress. Common asterisks (*) in the box plots indicate a tendency for significance (0.05 < *p* ≤ 0.1, Tukey HSD). Outliers are shown as dots. *n* = 6 per treatment.

**Figure 10:**
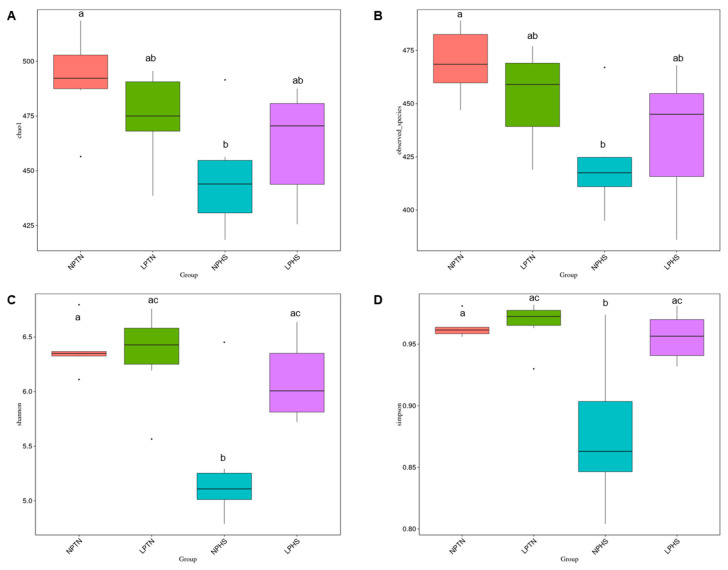
Alpha diversity of cecal bacterial community in broilers fed with low-protein diets during experimentally induced heat stress. Chao1 (**A**), Observed (**B**), Shannon (**C**), and Simpson (**D**). NPTN: normal-protein diet under thermoneutral; LPTN: low-protein diet under thermoneutral; NPHS: normal-protein diet under heat stress; LPHS: low-protein diet under heat stress. Different letters in the box plots indicate significant differences (*p* ≤ 0.05, Tukey HSD). *n* = 6 per treatment.

**Figure 11:**
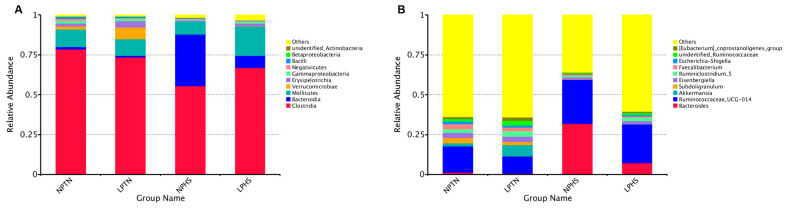
The composition of cecal bacterial populations in broilers fed with low-protein diets during experimentally induced heat stress. The relative abundance of bacterial community composition at the phylum level (**A**) and at the genus level (**B**). NPTN: normal-protein diet under thermoneutral; LPTN: low-protein diet under thermoneutral; NPHS: normal-protein diet under heat stress; LPHS: low-protein diet under heat stress. For clarity reasons, only the top 10 phyla and genera are shown. *n* = 6 for each treatment.

The beta diversity of the cecal bacterial community was analyzed by principal coordinate analysis (PCoA) of unweighted UniFrac distances, representing the diversity of cecal bacterial populations across all birds assigned to four treatments (Figure 9A), unweighted UniFrac distances shown across dietary groups (Figure 9B), PCoA of weighted UniFrac distances, representing the diversity of cecal bacterial populations across all birds assigned to four treatments (Figure 9C), and weighted UniFrac distances shown across dietary groups (Figure 9D). PCoA showed a tendency for separation and clustering when NPTN and NPHS were compared using unweighted UniFrac (*p* = 0.07; Figure 9A), suggestive of the differences in cecal microbiota composition among birds assigned to these groups (Figure 9B). Cecal microbial composition showed no clustering among groups when weighted UniFrac was considered (Figure 9C,D). The alpha diversity of the cecal bacterial community in each sample is shown in Figure 10A–D. The Chao, Observed_species, Shannon, and Simpson diversities of the cecal bacterial community were significantly different between NPTN and NPHS (Figure 10A–D). Further, the Shannon and Simpson diversities were significantly different between NPHS and LPHS (Figure 10C,D). Overall, the three main phyla present in all four dietary treatments were Clostridia, Mollicutes, and Bacteroidia (Figure 11A and Figure S3). At the genus level, *Ruminococcacea_UCG-014*, *Bacteroides*, and *Akkermansia* were the most abundant communities across all dietary treatments (Figure 11B and Figure S3).

**Figure 12:**
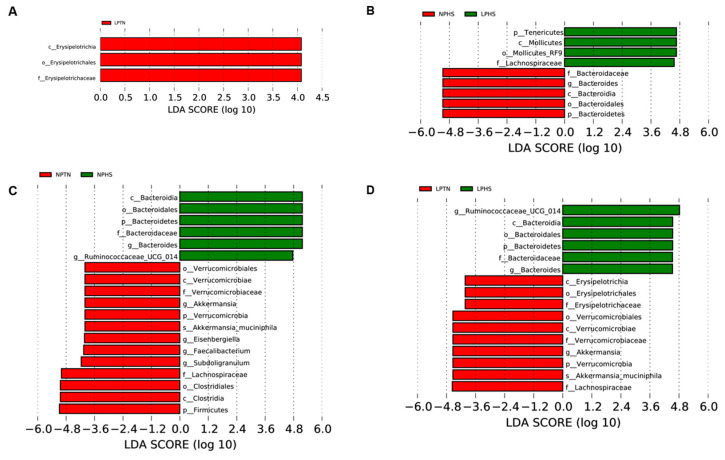
Histograms of cecal microbiota composition at the phylum level in broilers fed with low-protein diets during experimentally induced heat stress. Histograms of cecal microbiota composition using linear discriminant analysis (LDA) with effect size (LEfSe) for NPTN vs. LPTN (**A**), NPHS vs. LPHS (**B**), NPTN vs. NPHS (**C**), and LPTN vs. LPHS (**D**). NPTN: normal-protein diet under thermoneutral; LPTN: low-protein diet under thermoneutral; NPHS: normal-protein diet under heat stress; LPHS: low-protein diet under heat stress. *n* = 6 per treatment.

Linear discriminant analysis (LDA) with effect size measurements (LEfSe) was used to identify organisms that were different among dietary treatments. Compared to NPTN, the cecal contents of LPTN had higher proportions of *c_Erysipelotrichia*, *o_Erysipelotrichales*, and *f_Erysipelotrichaceae* (LDA [log10] score > 2.0; Figure 12A). The cecal contents of birds in the LPHS group were more enriched in *p_Tenericutes*, *c_Mollicutes*, *c_Mollicutes_RF9*, and *f_tachnospiraceae* compared to those in the NPHS treatment, while NPHS had a higher abundance of bacteria under the phylum Bacteroidetes (Figure 12B). Relative to the NPTN group, birds subjected to the NPHS treatment had higher proportions of *c_Bacteroidia*, *o_Bacteroidales*, *p_Bacteroidetes*, *f_Bacteroidaceae*, and *g_Bacteroides*, while the NPTN group had higher proportions of *o_Verrucomicrobiales*, *c_Verrucomicrobiae*, *f_Verrucomicrobiaceae*, and *g_Akkermansia* (Figure 12C). Lastly, compared to LPTN, the cecal contents of birds in the LPHS treatment group had higher proportions of *g_Ruminococcaceae_UCG_014*, *c_Bacteroidia*, *o_Bacteroidales*, *p_Bacteroidetes*, *f_Bacteroidaceae*, and *g_Bacteroides*, while the cecal contents of LPTN birds were more enriched in *c_Erysipelotrichia*, *o_Erysipelotrichales*, *f_Erysipelotrichaceae*, *o_Verrucomicrobiales*, *c_Verrucomicrobiae*, *f_Verrucomicrobiaceae*, and *g_Akkermansia* (Figure 12D).

## 3. Discussion

Low-protein diets reduce the FI, heat production, and mortality of broilers under HS [3,7,8]; however, these diets induce hyperphagia [9,10,11] and thermogenesis [10,12,13,14,15] under TN. The objective of this study was to investigate whether LP diets differentially regulate FI and thermogenesis during TN and HS conditions. Our study revealed several key findings: (1) While the LP group had a numerically higher FI than NP under TN conditions, the LP diet tended to reduce the FI during HS. This could be due to a lower mRNA abundance of duodenal ghrelin and higher GIP in the LP group during HS, (2) the LP diet increased the thermal radiation during TN but reduced it during HS, which might be explained by a higher mRNA abundance of muscle β1AR and AMPKα1 during TN, but not HS. Further, LP increased the mRNA abundance of COX IV during TN but reduced the COX IV and sirtuin 1 abundance during HS, (3) birds under HS had reduced FI and thermal radiation, which could be related to a higher transcript of ileum PYY and duodenal CCK and a decreased mRNA abundance of muscle β1AR, AMPKα1, and sirtuin 1, (4) chickens fed with the LP diet had higher abundances of cecal *p_Tenericutes*, *c_Mollicutes*, *c_Mollicutes_RF9*, and *f_tachnospiraceae* under HS, (5) under TN, the LP diet reduced plasma tyrosine and tryptophan. However, under HS, the LP group increased plasma tyrosine and normalized the tryptophan. In summary, our data provide evidence that LP diets mitigate the negative outcome of heat stress on the survivability of birds by reducing FI and heat production, which are regulated through factors expressed in the gut and skeletal muscle and changes in gut microbiota and plasma metabolomics.

In this study, birds fed with LP diets had a numerically higher FI than NP under TN conditions. LP-induced hyperphagia has been previously reported in birds [9,10,11,28]. However, the LP diet tended to reduce the FI during HS, which is in agreement with other published reports [3,7,28]. In contrast, other groups showed a reduction in FI in LP-fed birds during TN [29,30] or even no effect of LP diets on FI [31]. The discrepancy among studies could be due to differences in the level of protein restriction, the balance of limiting amino acids supplemented in the diet, and the type of the protein used. The LP-induced hyperphagia and “protein leverage” hypothesis has been described in several species, including birds [13,32,33]. According to this hypothesis, when animals are fed with LP diets, their total FI is increased as a strategy to avoid protein deficiency and meet the protein requirements. In the current study and most cited articles in this manuscript, the protein requirements of birds are defined based on total protein concept and excreta level, while the cecal fermentation may significantly modify protein digestion and amino acid digestibility. Therefore, testing the “protein leverage” hypothesis when the ileal digestibility of amino acids is considered would provide further insights into this phenomenon in poultry. Gut peptides play an important role in the regulation of FI in poultry [17,18,19]. LP diets have been shown to reduce the immunoreactive GLP-1 cells in the ileum of broilers under TN [20]. GLP-1 is considered a satiety peptide in mammals [34]. Likewise, GLP-1 significantly decreased the FI when injected intraperitoneally in Japanese quail [35]. The decreased FI of the LP group under HS could be due to a lower mRNA abundance of duodenal ghrelin and a higher GIP. The orexigenic effect of ghrelin has been previously reported in birds [36]. As GIP stimulates L-cells to secrete GLP-1 in mammals [37,38], it is likely that LP-induced hypophagia in HS is controlled by higher GLP-1 secretion. Further research is needed to better understand the role of peripheral and central signaling mechanisms in the differential regulation of FI under TN and HS when birds are fed protein-restricted diets.

Birds fed with LP diets under TN conditions had a higher thermal radiation than NP-fed birds while under HS conditions, LP birds had a lower thermal radiation than NP-fed birds. Similarly, others reported induced thermogenesis under TN [10,12,13,14,15] and decreased heat production during HS [8] in birds fed with the LP diet. We and others reported increased thermogenesis in response to protein and amino acid restriction during TN conditions in rodents [39,40,41,42] and pigs [43,44] as well. The differential effect of LP on diet-induced thermogenesis during TN and HS could be associated with changes in FI during TN and HS periods. Higher food consumption during TN and a lower intake during HS may contribute to variations in energy expenditure, as seen in this study and other works. The role of skeletal muscle in the regulation of thermogenesis in birds is evident [17]. Increased thermal radiation in birds fed with the LP diet during TN conditions may be associated with a greater transcript of muscle β1AR, AMPKα1, and COX IV. LP-fed birds had a decreased mRNA abundance of muscle sirtuin 1 and COX IV under HS, which might explain the lower thermal radiation of these birds during HS. Thermoregulation is controlled by the β-adrenergic hormonal system and AMPKα1 of skeletal muscle in avian species [45,46,47]. Other studies demonstrated that LP diets increase the expression of avUCP and adenine nucleotide translocase in the skeletal muscle of boilers under TN conditions [11,15]. Furthermore, increased heat production in broilers fed with LP diets under TN conditions has been linked with a deficiency of essential amino acids and an increased plasma T3 concentration [48,49]. We recently reviewed several mechanisms by which LP diets regulate energy expenditure in mammals, including sympathetic flux to brown adipose tissue and FGF21 and serotonergic signaling [50]. Due to the absence of brown adipose tissue in birds [51,52], further research is needed to explore the mechanisms of diet-induced thermogenesis in avian species. LP reduced the lean mass and lean percent and increased the fat mass and fat percent under both TN and HS conditions. A high body fat yield in broilers fed LP diets has been reported by others [9,10,11,15,53,54]. Compared to the NP diet, the LP-fed group, in an attempt to meet their protein needs, had relatively higher FI, and hence, their total energy intake from carbohydrates and fats was also increased. The extra energy consumed can be used either to store fat or increase heat production [13,55].

The three main phyla found in cecal contents were Clostridia, Bacteroidia, and Mollicutes. At the genus level, *Ruminococcacea_UCG-014*, *Bacteroides*, and *Akkermansia* were the most abundant communities. Similarly, in other studies, Clostridia and Bacteroidia were among the most prevalent bacterial species in the fecal microbiota of broilers [56,57]. Clostridium and Bacteroides have polysaccharide-degrading enzymes and can be involved in the degradation of non-starch polysaccharides found in cereal grains such as corn, which make up a large component of poultry diets [57,58]. Mollicutes were one of the major bacteria found in a higher abundance in LPHS compared to NPHS. Mollicutes have been associated with increased body fat and metabolic pathways related to the fermentation of simple carbohydrates and glycans [59]. Mollicutes metabolize carbohydrates to readily available short-chain fatty acids for absorption by the host [59]. Relative to the LPTN and NPHS treatments, LPHS had higher populations of Bacteroidia and Mollicutes, which is suggestive of increased metabolism and digestibility of non-starch polysaccharides and the uptake of simple sugars by LPHS. Therefore, it appears that LP diets increased the population of bacteria that digest carbohydrates under HS conditions. Metabolic pathway enrichment analysis showed that metabolites involved in carbohydrate, lipid, protein, and amino acid metabolism were affected by dietary protein content under TN and HS. In particular, several amino acids showed differential changes during TN and HS under protein restriction, which work as a signal to influence FI and energy expenditure. Several studies have reported that an increased concentration of circulatory amino acids such as glutamine, arginine, lysine, and methionine may induce the secretion of satiety peptides such as GLP-1 [21,60,61]. In this study, under TN, the LP diet reduced plasma tyrosine and tryptophan; however, under HS, the LP group increased plasma tyrosine and normalized the tryptophan. There is evidence that reduced dietary tyrosine and tryptophan are associated with increased FI and thermogenesis in rodents [62,63]. It remains to be determined whether alterations in plasma tryptophan and tyrosine contribute to the differential effects of LP diets on energy balance.

Heat stress is a major environmental concern that not only affects the birds’ immunity, metabolism, and growth performance but also has adverse effects on FI and body composition. For every 1 °C increase in environmental temperature, FI decreases by 3.6% in broilers [64]. Birds under HS had reduced FI in the current study, which could be related to the higher transcript of ileum PYY and duodenal CCK. In our study, overall HS reduced thermal radiation, which could be linked with the reduced mRNA expression of β1AR, AMPKα1, and sirtuin 1. Oxidative stress may occur during HS due to the increased levels of reactive oxygen species that cannot be neutralized by limited levels of natural antioxidants [65]. These reactive species can result in extensive tissue and cellular damage, causing major physiological and behavioral responses, which contribute to reducing productivity, immunity, nutrient digestibility, and meat quality [66]. The plasma MDA concentration was higher during HS in the current study. Likewise, others found that serum and liver MDA concentration was increased under HS [67,68]. This is suggestive of an increased lipid peroxidation during HS and reflects an indirect overproduction of reactive oxygen species in the body. During HS, birds had a lower BMC, BMC percent, BMD, lean mass, fat mass, and fat percent.

## 4. Materials and Methods

### 4.1. The Animals and Housing

All the experimental procedures performed in this study were approved by Oklahoma State University’s Institutional Animal Care and Use Committee (Animal Care and Use Protocol # AG-19-5). A total of 200 day-old male Cobb × Cobb 500 broiler chicks were obtained from Cobb commercial hatchery (Siloam Springs, AR, USA) and housed in a closed building with no windows and with concrete floor pens covered with wood shavings, single-hole stainless steel feeders, and nipple drinkers. The building was equipped with central heating, cooling, and ventilation systems. The ambient temperature and lighting program was in accordance with breeding company recommendations. Briefly, the room temperature was set at 32 °C, 29 °C, 27 °C, 25 °C, 22 °C, and 20 °C for days 0–7, 8–14, 15–21, 22–28, 29–35, and 36–42, respectively. The light/dark periods were 23:1, 16:8, 17:7, 18:6, 19:5, 20:4, 21:2, 22:2, and 23:1 on days 0–5, 6–22, 23, 24–36, 37, 38, 39, 40, and 41–42, respectively, with an intensity of 20 lux during the light period. The light source used was LED bulbs. The range of relative humidity was 50 to 65% in the building.

### 4.2. Diets, Experimental Design, and Heat Stress Protocol

Upon arrival, chicks (*n* = 200) were randomly assigned into 36 pens with an average of 5 to 6 chicks per pen. The average stocking density for birds was 12.5 kg/m^2^. After a 2-week acclimation period (i.e., starter phase), birds were weight-matched (472.2 ± 39.0 g) and subjected into four groups (9 pens/group) for 4 weeks, including the NPTN, LPTN, NPHS, and LPHS. The starter, grower, and finisher phase diets (2 weeks each) were prepared as recommended by Nutritional Requirements of Poultry by the National Research Council [69]. The ingredients and composition of the diets are shown in Table 5. The crude protein (CP) level of LP diets was obtained by reducing the soybean meal, and these diets were supplemented with limiting amino acids (i.e., lysine, methionine, threonine, tryptophan, isoleucine, valine, and glycine) equal to the normal-protein diets. Normal-protein and LP diets were isocaloric, which was achieved by adjusting the corn levels after dropping the soybean for LP diets. Birds were fed once a day at 6 pm and had *ad libitum* access to both feed and water throughout the study. The study was carried out in the same building in 2 consecutive experiments. In experiment 1, birds in the HS treatment experienced a cyclic HS at 35 °C for 7 h/day as reported by others [70] from 11 am to 6 pm. In experiment 2, broilers assigned to the TN treatment were housed under ambient temperature, according to breeding company recommendations as described earlier.

### 4.3. Behaviors and Physiological Adaptations Measurements

During the daily HS period, i.e., 11 am–6 pm, the behaviors and physiological adaptation measurements of birds were recorded at three separate phases: 11 am–1 pm, 2–4 pm, and 4–6 pm. Behavioral observations included the percentage of birds eating, drinking, wing spreading, and panting in each pen. Physiological observations recorded were respiratory rate (breaths/minute) and rectal temperature of randomly selected broilers in each pen. The respiratory rate was recorded visually by one individual throughout the experiment, and rectal temperature was measured using a digital thermometer. During these observations, pen temperature and room temperature/humidity were recorded using a humidity/temperature chart recorder data logger and a digital weather station, respectively.

### 4.4. Food Intake and Growth Measurements

Food intake and water intake were recorded daily at 6 pm. Pen body weight was measured weekly and divided by the number of birds in each pen. Based on these records, the ADG, ADFI, ADWI, G:F, and W:F were calculated. Furthermore, based on the analyzed concentration of CP% in diets, the ADPI and G:P were calculated.

### 4.5. Thermal Images

Thermal images were captured from one randomly selected bird in each pen during both TN and HS periods between 11 am and 6 pm using an FLIR C2 compact thermal camera with a focal length of 1.54 mm and a thermal accuracy of ±2 °C (FLIR Systems, Boston, MA, USA). The emissivity coefficient was set at 0.86. The camera was positioned roughly 1 m above each pen, and each image included at least one bird positioned in the center of the image.

### 4.6. Feed Samples Collection

Feed samples were collected during mixing diets. A feed sample (~50 g) was collected from each feed bag and pooled for each diet phase, and subsamples were collected for composition analysis. Feed samples were stored at −20 °C until proximate analysis for feed composition.

### 4.7. Blood and Tissue Samples Collection

At the end of the finisher phase, one bird was selected randomly in each pen and euthanized via CO_2_ asphyxiation. Tissue samples including pectoralis major, duodenum, ileum, and cecal content were collected. All tissues and cecal contents collected were wrapped in foil, snap-frozen in liquid nitrogen, and stored at −80 °C until further analysis. Blood samples were collected from 1 randomly selected bird/pen (i.e., 8–9 chickens from each treatment). Blood samples were collected in pre-labeled 3 mL EDTA-coated blood collection tubes (BD Vacutainer^®^, Franklin Lakes, NJ, USA) from the jugular anterior vena cava after euthanasia. Blood tubes were immediately placed on ice after collection, transferred to the lab, centrifuged at 2000× *g* for 10 min at 4 °C, and plasma was separated. The collected plasma was stored at −80 °C until further analysis.

### 4.8. Body Composition Analysis

At the end of the study, 1 bird per pen was randomly selected and euthanized as described above. Following euthanasia, birds were scanned by dual-energy X-ray absorptiometry (DEXA) (Hologic, Discovery QDR series, Bedford, MA, USA) to determine the BMD, BMC, lean mass, and fat mass, following published protocols [71,72].

### 4.9. Radiative Heat Transfer Analysis

Thermal images were analyzed using the FLIR Research Studio 2.1 (FLIR Systems, Boston, MA, USA). To obtain the dorsal surface body mean temperature, a region of interest was drawn in a circle shape on the back of the bird (Figure S4). The radiative heat transfer of the birds (W/m^2^) was calculated using the following equation: $\sigma \varepsilon \left(T_{s}^{4}-T_{a}^{4}\right)$ [73,74], where *σ* is Stefan-Boltzmann constant (5.67 × 10^−8^ W/m^2^K^4^), *ε* is thermodynamic emissivity (0.86), *T_s_* is body surface temperature (kelvin), and *T_α_* is the ambient temperature (kelvin).

### 4.10. Diet Composition Analysis

Diet composition analyses were performed by ServiTech (Dodge City, KS, USA) for dry matter, CP, crude fat, crude fiber, calcium, and phosphorus, as we previously described [71,75].

### 4.11. Reverse Transcription Polymerase Chain Reaction (RT-qPCR)

RNA isolation and RT-qPCR was performed for CCK, ghrelin, GIP, secretin in duodenum, PYY in ileum, β1AR, AMPKα1, sirtuin 1, COX IV, and PGC-1α in the breast muscle, following our published procedures [76,77,78]. After isolation, the RNA concentration and the ratio of absorbance at 260 and 280 nm (260:280) were measured by Nanodrop spectrophotometer (Nanodrop^®^ Technologies, Wilmington, DE, USA), and only samples with 260:280 ratios of 1.9–2.1 nm were used for analysis. Complementary DNA was synthesized in a T100™ Thermal Cycler (Bio-Rad, CA, USA). The sequences for primers were obtained from other publications [45,79,80,81,82,83,84] and cross-checked using the NCBI Primer-BLAST tool (https://www.ncbi.nlm.nih.gov/tools/primer-blast/, accessed on 5 February 2020). The sequences of primers used and other details are listed in Table S3. Using a CFX96 real-time PCR detection system (Bio-Rad, Hercules, CA, USA), the cycle threshold (Ct) values for target genes and β-actin, as a reference gene, were obtained, and melt curve analysis was performed, as we previously described [76,77]. The relative abundance of target genes was determined using the 2^−ΔΔCt^ method.

### 4.12. Plasma Oxidative Stress Biomarkers

SOD activity, glutathione peroxidase (GPx) activity, lipid peroxidation of MDA, and LPO in plasma were measured by using SOD Assay Kit (ab65354), Glutathione Assay Kit (ab102530), MDA Assay Kit (ab118970), and LPO Assay Kit (ab133085), respectively, according to the manufacturer’s instructions (Abcam, Cambridge, MA, USA). The optical density for SOD, GPx, MDA, and LPO kits was read at 450, 340, 532, and 500 nm wavelengths, respectively, using an Epoch microplate spectrophotometer (BioTek^®^ Instruments, Inc. Highland Park, VT, USA). The intra-assay CV for SOD, GPx, MDA, and LPO was 2.9%, 0.1%, 1.3%, and 1.1%, respectively. The inter-assay CV for SOD and GPx was 2.7% and 0.1%, respectively.

### 4.13. Plasma Metabolomics

Plasma metabolomics analysis was performed at the West Coast Metabolomics Center (UC Davis, Davis, CA, USA), as we previously described [43,44]. Briefly, following sample preparations, plasma samples were analyzed by gas chromatography (GC)—mass spectrometry (MS) using a time-of-flight mass spectrometer (GC-TOF MS; Leco Pegasus IV, USA). An Agilent 690 GC was equipped with an automatic linear exchange (ALEX; Gerstel Corporation, Linthicum, MD, USA) and cold injection system (CIS; Gerstel corporation, Linthicum, MD, USA) for data acquisition. Following data acquisition and processing, the values for quantified metabolites were reported as the peak height. The metabolomics data analysis was performed using MetaboAnalyst 3.092 (available online at: http://www.metaboanalyst.ca/faces/ModuleView.xhtml, accessed on 20 January 2021). A PCA, pathway impact analysis, and hierarchical clustering analysis were performed.

### 4.14. Cecal Contents Microbiome

Cecal content DNA isolation, amplicon sequencing, sequence data analysis, and taxonomic classification were performed, following our previously published protocols [71]. PCR amplification, microbial amplicon sequencing, and bioinformatics were completed at Novogene Corp. (Sacramento, CA, USA). Briefly, after DNA isolation from cecal contents, amplifying the16S rRNA V4 region by PCR and sequencing library preparation, the Illumina HiSeq 2500 platform (Illumina, Inc., San Diego, CA, USA) was used to sequence the library. Following the sequence data analysis, taxonomic classification was performed, and rarefaction curves were generated. The beta diversity of bacterial populations was assessed by PCoA and weighted and unweighted UniFrac methods. Furthermore, LDA with LEfSe was used for the quantitative analysis of cecal microbiota composition within experimental groups.

### 4.15. Statistical Analysis

For assessing the rate of mortality associated with HS and the effect of diet on it, a Log-Rank survival curve analysis was performed using SPSS 23 (IBM SPSS Statistics, Armonk, NY, USA). The percent of birds that were alive was calculated on a per to pen basis for each week of the experiment, which was then analyzed using a Mixed analysis procedure, as described below. The initial BW, final BW, ADFI, ADG, ADPI, ADWI, G:F, G:P, W:F, heat stress behavior observations (eating, drinking, panting, wing spread, respiratory rate, and rectal temperature), DEXA (BMC, BMD, lean mass, and fat mass), and qPCR data were analyzed using two-way ANOVA in the GLM procedure of SPSS 23 (IBM SPSS Statistics, Armonk, NY, USA), including diet, temperature, and the interaction of diet and temperature in the model. The growth performance related to the phase (grower and finisher), behavioral adaptations, and radiative heat transfer data were analyzed using a Mixed analysis procedure of SPSS 23 (IBM SPSS Statistics, Armonk, NY, USA). Diet, temperature, the phase, interaction of diet and temperature, diet and time, temperature and time, and diet, temperature, and time were included in the model as fixed effects. The chicken was the random variable. Based on the smallest values of fit statistics for corrected Akaike’s Information Criterion and Bayesian Information Criterion, the covariance structure of the repeated measurements for each variable was modeled as either first order antedependence, autoregressive, heterogeneous autoregressive, compound symmetry, heterogenous compound symmetry, or toeplitz. Oxidative stress biomarkers and the peak height of metabolites in plasma were analyzed using two-way ANOVA in the GLM procedure of SPSS 23 (IBM SPSS Statistics, Armonk, NY, USA), including diet, temperature, and the interaction of diet and temperature in the model. The paired *t*-test was used for post hoc analysis to detect the “diet” effect within the TN and HS groups. Differences were considered significant at *p* ≤ 0.05, and trends were considered at 0.05 < *p* ≤ 0.10.

## 5. Conclusions

The differential effect of LP diets on food intake and heat production during TN and HS is regulated by peripheral factors expressed in the gut and skeletal muscle. Furthermore, protein restriction differentially influenced the plasma metabolomics and cecal microbiota composition during TN and HS, which may contribute to the metabolic outcome of protein dilution. Whether other peripheral and central mechanisms are involved in sensing a protein deficiency during TN and HS conditions in avian species remains poorly understood.

## Figures and Tables

**Table 1:** Behavioral adaptations of broilers fed with low-protein diets during experimentally induced heat stress.

Items	Treatments ^1^				SEM ^2^	*p*-Values		
	NPTN ^2^	LPTN ^2^	NPHS ^2^	LPHS ^2^		Diet	Temp ^2^	Diet × Temp
Eating (%)	9.0	10.2	3.0	3.6	0.6	0.13	≤0.01	0.64
Drinking (%)	8.2	5.6 *	8.1	6.8 *	0.3	≤0.01	0.17	0.14
Panting (%)	1.8	3.5 *	87.2	86.5	7.3	0.61	≤0.01	0.24
Wing spread (%)	0.0	0.0	27.4	28.3	2.5	0.72	≤0.01	0.72
Respiratory rate (breath/min)	65.2	67.0 ^#^	199.5	199.8	11.6	0.34	≤0.01	0.52
Rectal temperature (°C)	40.3	40.4	42.0	42.2	0.2	0.28	≤0.01	0.79

^1^ The values are the mean; *n* = 9 pens (5–6 birds/pen). ^2^ NPTN: normal-protein diet under thermoneutral; LPTN: low-protein diet under thermoneutral; NPHS: normal-protein diet under heat stress; LPHS: low-protein diet under heat stress; SEM: standard error of means; Temp: temperature. * Within rows, NPTN vs. LPTN and NPHS vs. LPHS *p* ≤ 0.05. ^#^ Within rows, NPTN vs. LPTN and NPHS vs. LPHS 0.05 < *p* ≤ 0.10.

**Table 2:** Growth measurements of broilers fed with low-protein diets during experimentally induced heat stress.

Items	Treatments ^1^				SEM ^2^	*p*-Values		
	NPTN ^2^	LPTN ^2^	NPHS ^2^	LPHS ^2^		Diet	Temp ^2^	Diet × Temp
Initial BW ^3^, g	471	516	548	553	10	0.14	0.38	0.21
Final BW ^3^, g	2737	2688	2185	1901	65	0.21	0.22	0.13
ADG ^3^, g/d	78	74	54	44 *	3	≤0.01	≤0.01	0.11
ADFI ^3^, g/d	169	181	158	140 ^#^	4	0.63	≤0.01	0.03
ADPI ^3^, g/d	36	27 *	24	20 ^#^	1	≤0.01	≤0.01	0.02
ADWI ^3^, mL/d	319	241 *	373	298 *	9	≤0.01	≤0.01	0.76
G:F ^3^, g/g	0.5	0.4 *	0.3	0.3	0.01	0.11	0.06	0.47
G:P ^3^, g/g	2.5	3.0 *	4.3	4.0	0.19	0.51	0.03	≤0.01
W:F ^3^, mL/g	1.9	1.3 *	2.4	2.2	0.08	≤0.01	≤0.01	0.05

^1^ The values are the mean; *n* = 9 pens (5–6 birds/pen). ^2^ NPTN: normal-protein diet under thermoneutral; LPTN: low-protein diet under thermoneutral; NPHS: normal-protein diet under heat stress; LPHS: low-protein diet under heat stress; SEM: standard error of means; Temp: temperature. ^3^ BW: body weight; ADG: average daily gain; ADFI: average daily food intake; ADPI: average daily protein intake; ADWI: average daily water intake; G:F: gain: food; G:P: gain: protein; W:F: water: food. * Within rows, NPTN vs. LPTN and NPHS vs. LPHS *p* ≤ 0.05. ^#^ Within rows, NPTN vs. LPTN and NPHS vs. LPHS 0.05 < *p* ≤ 0.10.

**Table 3:** Bone and body characteristics of broilers fed with low-protein diets during experimentally induced heat stress.

Items	Treatments ^1^				SEM ^2^	*p*-Values		
	NPTN ^2^	LPTN ^2^	NPHS ^2^	LPHS ^2^		Diet	Temp ^2^	Diet × Temp
BMC ^3^ (g)	35.40	34.14	26.09	24.04	1.10	0.21	≤0.01	0.76
BMC (%)	1.18	1.24	1.09	1.12	0.02	0.27	0.02	0.65
BMD ^3^ (g/cm^2^)	0.15	0.15	0.14	0.14	0.002	0.97	0.04	0.17
Lean mass (g)	2747.85	2526.96 *	2262.61	1983.51 *	64.47	≤0.01	≤0.01	0.76
Lean (%)	94.40	88.00 *	94.33	92.25 *	0.65	≤0.01	≤0.01	≤0.01
Fat mass (g)	134.79	282.53 *	109.64	142.51 *	14.72	≤0.01	≤0.01	≤0.01
Fat (%)	4.46	9.96 *	4.57	6.64 *	0.64	≤0.01	≤0.01	≤0.01

^1^ The values are the mean; *n* = 8. ^2^ NPTN: normal-protein diet under thermoneutral; LPTN: low-protein diet under thermoneutral; NPHS: normal-protein diet under heat stress; LPHS: low-protein diet under heat stress; SEM: standard error of means; Temp: temperature. ^3^ BMC: Bone mineral content; BMD: Bone mineral density. * Within rows, NPTN vs. LPTN and NPHS vs. LPHS *p* ≤ 0.05.

**Table 4:** Significantly different plasma metabolites in broilers fed with low-protein diets during experimentally induced heat stress.

Metabolites	Treatments ^1^				SEM ^2^	*p*-Values		
	NPTN ^2^	LPTN ^2^	NPHS ^2^	LPHS ^2^		Diet	Temp ^2^	Diet × Temp ^2^
**Microbiome Metabolism**								
Daidzein	981	456	450	292 ^#^	77	0.11	≤0.01	0.57
Dehydroabietic acid	15,535	11,050	39,862	22,649 *	2268	0.04	≤0.01	0.02
D-erythro-sphingosine	3241	1570 ^#^	4367	2602	274	0.05	≤0.01	0.57
Lanosterol	691	722 *	437	615 *	44	≤0.01	0.06	0.99
Lyxitol	67,091	80,787 *	256,403	180,839	16,853	0.99	≤0.01	0.02
**Carbohydrate Derivatives**								
Azelaic acid	4516	954 *	1010	2492 *	420	0.45	0.26	≤0.01
Conduritol-beta-epoxide	101,941	35,050 *	47,753	19,999 *	6070	≤0.01	≤0.01	0.09
Galactinol	27,915	4352 ^#^	2314	4083	3215	0.08	0.02	0.03
Gluconic acid	13,217	7666	16,148	7308 *	1067	≤0.01	0.25	0.05
Hypoxanthine	104,405	74,054	138,897	146,782	9351	0.23	≤0.01	0.45
Inosine	18,380	12,763	40,730	49,878	3913	0.16	≤0.01	0.22
Inulotriose	1236	382	236	313 ^#^	134	0.29	0.02	0.11
Maltotriose	1938	870	470	600	190	0.52	≤0.01	0.23
Melezitose	1386	413 ^#^	276	398	137	0.19	0.02	0.04
Mucic acid	6005	1306 *	1860	1287	519	≤0.01	≤0.01	0.02
Pinitol	131,195	14,550 *	25,924	14,371 *	13,666	≤0.01	0.02	0.03
Quinic acid	754	679 *	970	708 *	202	≤0.01	0.03	0.38
Ribonic acid	4072	2204 ^#^	4061	4129	279	0.82	0.03	0.24
Saccharic acid	45,410	6313 *	8514	6361	4327	≤0.01	≤0.01	≤0.01
Uridine	19,823	10,327	51,224	23,595 *	3337	0.02	≤0.01	0.08
Xylitol	85,529	35,367 ^#^	146,623	74,144 ^#^	9576	0.02	≤0.01	0.33
**Carbohydrates**								
Alpha-ketoglutarate	72,780	52,059	109,710	72,904 ^#^	5242	0.25	≤0.01	0.08
Anhydroglucitol	17,074	22,049 *	20,792	18,979	950	≤0.01	0.97	0.22
Arabitol	124,635	137,613 *	342,129	290,263	23,793	0.24	≤0.01	0.24
Ribose	79,755	14,043 ^#^	28,413	14,838 *	8831	0.03	0.18	0.15
Erythritol	102,640	76,671	482,304	169,023 *	32,002	≤0.01	≤0.01	≤0.01
Erythrose	1857	940	1811	980 *	115	≤0.01	0.50	0.42
Fructose-6-phosphate	2559	821 *	1221	1088	169	0.02	0.16	≤0.01
Galactitol	68,449	42,934	135,739	49,422 *	10,523	0.02	0.02	0.02
Glucose-1-phosphate	7286	3336	9567	4894 *	616	≤0.01	≤0.01	0.23
Glucose-6-phosphate	4009	1353 *	3123	2191	262	≤0.01	0.34	0.16
Lactose	30,940	11,331	9422	10,569	2704	0.21	0.02	0.06
Maltose	108,960	27,381 ^#^	14,205	24,420	11,347	0.15	≤0.01	0.03
Raffinose	134,815	11,567 *	2275	12,737	16,955	0.07	0.02	0.03
Ribitol	17,360	8404	47,434	16,659 *	3597	0.02	≤0.01	0.06
Sorbital	127,390	69,622	229,553	105,516 *	15,463	0.02	≤0.01	0.08
Sucrose	229,381	19,729 ^#^	2622	24,149	31,247	0.12	0.04	0.05
Tagatose	2062	1328	2371	1370 *	111	0.04	0.08	0.04
Threitol	13,480	10,000	24,489	14,506 *	1117	≤0.01	≤0.01	≤0.01
Xanthosine	805	518	1309	837	97	0.50	0.02	0.57
Xylose	35,808	28,893 *	36,246	31,959	1386	0.05	0.12	0.25
**Protein Metabolism**								
Creatinine	164,384	22,548 *	161,980	47,249 *	14,773	≤0.01	0.14	0.94
Ornithine	237,847	52,406 *	295,108	49,267 *	20,748	≤0.01	≤0.01	≤0.01
Urea	269,631	74,397 *	216,172	45,945 *	20,909	≤0.01	0.29	0.61
Uric Acid	448,970	204,713	698,371	192,455 *	47,905	≤0.01	0.02	≤0.01
Citrulline	77,311	15,647 *	79,028	16,167 *	6222	≤0.01	0.42	0.36
Xanthine	20,784	11,974	31,289	21,351	2161	0.48	≤0.01	0.76
**Amino Acids**								
Alanine	973,266	2,008,637 *	725,231	788,927	117,331	≤0.01	≤0.01	≤0.01
Asparagine	68,949	116,533 *	58,051	87,093 *	5217	≤0.01	0.08	0.08
Aspartic Acid	332,966	156,243 *	262,639	169,632 ^#^	15,225	≤0.01	0.93	0.58
Cyanoalanine	1040	2143 *	612	1741 *	135	≤0.01	0.07	0.41
Cysteine	12,932	21,167 *	15,309	24,415 *	1342	≤0.01	0.12	0.83
Cystine	92,902	102,225 *	101,022	122,176 *	3516	≤0.01	0.06	0.81
Glutamine	1,543,054	4,304,606 *	3,263,465	4,764,719 *	294,103	≤0.01	0.17	0.15
Glycine	2,009,209	3,528,145 *	864,965	1,947,990 *	193,982	≤0.01	≤0.01	≤0.01
Histidine	119,164	76,831	148,762	52,758 *	8802	≤0.01	0.63	≤0.01
Homocystine	3018	2565	5747	2782 ^#^	395	0.27	0.04	0.03
Homoserine	1206	2145 *	525	1785 *	130	≤0.01	≤0.01	0.81
Isoleucine	952,169	997,787 *	875,481	709,461	31,524	≤0.01	0.05	≤0.01
Leucine	2,153,375	1,601,125	1,888,973	1,308,442 ^#^	69,567	0.86	0.14	0.05
Lysine	49,913	115,908 *	138,591	49,868 *	9792	0.42	0.87	≤0.01
Methionine	268,057	292,729 *	145,867	248,519 *	14,091	≤0.01	≤0.01	0.90
Oxoproline	1,529,819	1,806,087 *	2,423,289	4,983,321 *	292,809	≤0.01	≤0.01	0.03
Proline	602,393	622,686 *	403,365	419,135	24,679	≤0.01	≤0.01	0.06
Serine	3,638,367	8,429,353 *	2,420,920	6,817,181 *	483,269	≤0.01	0.06	0.30
Threonine	1,363,373	4,080,860 *	278,928	2,330,931 *	281,695	≤0.01	≤0.01	0.07
Tryptophan	632,209	529,272 *	601,717	472,079	20,849	0.20	0.41	0.02
Tyrosine	1,687,441	1,592,337 *	760,553	936,369 *	88,889	≤0.01	≤0.01	0.33
Valine	839,510	641,914 ^#^	670,200	567,541	23,844	0.13	0.02	0.12
**Fatty Acid Metabolism**								
2-aminobutyric acid	22,894	51,013 *	28,548	33,046 ^#^	2581	≤0.01	0.05	≤0.01
2-deoxytetronic acid	6335	3268 *	6897	5090	355	0.03	≤0.01	0.82
2-hydroxyglutaric acid	16,007	7221	52,619	19,386 *	3798	≤0.01	≤0.01	0.04
2-hydroxyvaleric acid	12,376	84,595	15,595	8457 *	739	≤0.01	0.03	≤0.01
3-hydroxybutyric acid	906,092	783,907	1,457,671	740,685 *	90,306	0.72	≤0.01	0.92
Beta-sitosterol	10,643	5324	5284	5139	592	0.41	≤0.01	0.10
Cholesterol	4,343,379	3,459,489 ^#^	3,347,360	3,539,811 ^#^	175,985	≤0.01	0.39	0.72
Glycerol	1,517,631	714,810	3,270,495	1,716,658	244,585	0.11	≤0.01	0.37
Glycerol-alpha-phosphate	8362	3151 *	10,609	5407 ^#^	681	≤0.01	≤0.01	0.51
Lauric acid	27,298	24,131 ^#^	19,406	17,229	1231	0.08	≤0.01	0.09
Linoleic acid	5170	4769	3030	2728	391	0.21	≤0.01	0.37
Monostearin	1149	817	661	684	78	0.37	0.07	≤0.01
Palmitoleic acid	1475	1349 *	2359	2237	204	0.18	≤0.01	0.93
**Amino Acid Metabolism**								
3-hydroxyanthralinic acid	755	1316 *	989	580	97	0.06	0.11	≤0.01
Aminomalonate	173,601	301,913 *	63,777	164,296 *	18,198	≤0.01	≤0.01	0.03
Aminoisobutyric acid	7837	5210	4341	4472	465			
Fumaric acid	257,967	98,137	1,051,705	274,893 *	87,753	≤0.01	≤0.01	0.03
Guanidinosuccinate	1975	1478	1359	1406 *	71	≤0.01	≤0.01	0.60
Ketoisocaproic acid	35,336	25,286	29,942	15,621 ^#^	2043	0.18	0.06	0.05
**Amino Acid Derivatives**								
Cytosin	1575	920	2333	994 *	179	0.04	0.08	0.10
Dihydroxypyrazine	12,424	12,135 *	10,803	12,094 ^#^	359	≤0.01	0.98	0.56
5-hydroxynorvaline	6412	11,442 *	3516	9150 *	627	≤0.01	≤0.01	0.36
Maleimide	12,622	9896 ^#^	6841	8918 *	584	≤0.01	≤0.01	0.40
Malic acid	443,186	164,881	1,208,719	421,807 ^#^	100,106	0.03	≤0.01	0.16
Malonic acid	1616	662	542	448	131	0.11	≤0.01	0.16
Phosphoethanolamine	195,180	129,800 ^#^	114,912	147,009 *	376	0.01	0.22	0.67
Pimelic acid	7760	3957 ^#^	3794	7610 *	614	0.11	0.70	≤0.01
Pipecolinic acid	1964	1603 *	1041	1805 *	123	≤0.01	0.16	0.11
Serotonin	38,246	3197 *	8308	7403	4485	0.04	0.15	0.04
Spermidine	1623	1769 *	1350	2194 *	125	≤0.01	0.40	0.44
Trans-4-hydroxyproline	581,997	650,934 *	462,090	587,206 *	30,640	≤0.01	0.25	0.51
**Vitamins**								
Creatine	202,876	24,225 *	194,651	61,177 *	18,256	≤0.01	0.12	0.80
Isothreonic acid	31,145	28,587 *	44,912	38,834	1612	0.02	≤0.01	0.11
Methionine sulfoxide	46,777	50,248 *	33,994	54,009 *	2550	≤0.01	0.52	0.64
N-acetyl-D-galactosamine	4327	3112	9057	4913 ^#^	608	0.23	≤0.01	0.09
N-acetylglycine	2720	1839	916	1069	300	0.61	≤0.01	0.79
N-epsilon-trimethyllysine	5077	2024 *	2594	1728	304	≤0.01	≤0.01	0.09
Nicotinamide	7677	13,374 *	106,67	10,662	655	≤0.01	0.91	0.02
Pentitol	1914	927 *	954	587	110	≤0.01	≤0.01	0.54
Threonic acid	18,822	12,198	19,678	10,973 *	851	≤0.01	0.50	≤0.01
Tocopherol alpha	38,575	49,087 *	27,368	22,267	2628	≤0.01	≤0.01	≤0.01
Tocopherol gamma	2279	2942 *	1236	1220	172	≤0.01	≤0.01	≤0.01
**Lipids**								
Zymosterol	9932	8408 ^#^	5406	8045 *	619	≤0.01	0.07	0.37
**Miscellaneous**								
Butyrolactam	22,155	17,057	25,627	16,183 ^#^	1314	0.42	0.73	0.04
Dihydroxybutanoic acid 2-3	30,697	39,023 *	19,976	39,404 *	2054	≤0.01	0.13	0.56
Ethanolamine	159,642	114,369	185,066	136,941	8063	0.73	0.05	0.34
Phenylacetic acid	2038	3109 *	1674	2521 *	172	≤0.01	0.09	0.11
Pseudouridine	4679	2651	5355	3891	337	0.34	0.02	0.99
Succinic acid	907,060	509,673	2,894,350	982,356 *	201,883	≤0.01	≤0.01	0.02

^1^ The values are the mean peak height; *n* = 8 per treatment. ^2^ NPTN: normal-protein diet under thermoneutral; LPTN: low-protein diet under thermoneutral; NPHS: normal-protein diet under heat stress; LPHS: low-protein diet under heat stress; SEM: standard error of means; Temp: temperature. * Within rows, NPTN vs. LPTN and NPHS vs. LPHS *p* ≤ 0.05. ^#^ Within rows, NPTN vs. LPTN and NPHS vs. LPHS 0.05 ≤ *p* ≤ 0.10.

**Table 5:** Ingredients and nutrient contents of experimental diets ^1^ (As-fed basis).

Ingredients ^5^, %	Starter ^2^	Grower ^3^		Finisher ^4^	
		NP	LP	NP	LP
Corn, yellow dent	60.58	65.34	79.59	69.99	83.81
Soybean meal, 47.5% CP	33.05	28.94	13.26	25.04	9.58
Dicalcium phosphate 18.5%	2.09	1.71	2.12	1.50	1.98
Limestone	1.18	1.09	1.07	0.95	0.95
Salt	0.44	0.33	0.32	0.25	0.25
Choline Chloride 60%	0.12	0.08	0.08	0.03	0.03
Minerals and vitamins premix ^6^	0.40	0.40	0.40	0.37	0.40
Magnesium oxide	0.83	0.72	0.70	0.75	0.75
L-Lysine HCl	0.19	0.17	0.64	0.10	0.57
DL-Methionine	0.23	0.12	0.19	0.08	0.14
L-Threonine	0.17	0.16	0.37	0.15	0.36
L-Tryptophan	-	-	0.09	-	0.10
L-Isoleucine	0.07	0.06	0.05	0.05	0.06
L-Valine	0.10	0.09	0.11	0.05	0.12
Glycine	0.55	0.49	0.72	0.37	0.60
Chromium oxide	-	0.30	0.30	0.30	0.31
**Calculated Nutrient Content**					
Dry matter, %	88.98	88.93	88.70	88.97	88.73
ME, kcal/kg	3256	3270	3274	3284	3285
Crude protein, %	21.80	20.09	14.75	18.39	13.17
Crude fiber, %	2.52	2.49	2.28	2.46	2.25
Crude fat, %	3.35	3.42	3.50	3.48	3.56
L-Lysine, %	1.11	1.00	0.99	0.85	0.85
L-Threonine, %	0.80	0.73	0.73	0.68	0.68
DL-Methionine, %	0.50	0.38	0.38	0.33	0.32
L-Tryptophan, %	0.23	0.21	0.21	0.19	0.19
Glycine, %	1.25	1.14	1.14	0.97	0.97
L-Isoleucine, %	0.80	0.73	0.47	0.66	0.42
L-Valine, %	0.89	0.83	0.60	0.73	0.55
L-Leucine, %	1.50	1.42	1.06	1.34	0.99
L-Histidine, %	0.65	0.59	0.37	0.54	0.32
L-Arginine, %	1.21	1.09	0.66	0.99	0.56
L-Phenylalanine, %	0.87	0.80	0.53	0.74	0.47
Calcium, %	1.04	0.90	0.93	0.80	0.85
Phosphorus, %	0.79	0.70	0.71	0.65	0.67
**Analyzed Chemical Composition**					
Dry matter, %	87.00	87.60	87.20	86.80	87.10
Crude protein, %	18.50	19.40	14.10	17.20	12.70
Crude fat, %	2.40	2.30	2.50	2.50	2.60
Calcium, %	1.33	1.34	1.07	1.08	1.15
Phosphorus, %	0.83	0.73	0.78	0.69	0.67

^1^ NP: normal-protein diet (NRC, 1994) [69]; LP: low-protein diet. ^2^ Diets were provided from day 1 to 13. ^3^ Diets were provided from day 14 to 27. ^4^ Diets were provided from day 28 to 42. ^5^ Corn, soybean meal, dicalcium phosphate, limestone, salt, choline chloride, glycine, lysine, DL-methionine, and magnesium oxide were obtained from Nutra Blend, LLC (Neosho, MO, USA). L-threonine (98.5%) and L-tryptophan (98%) were obtained from Ajinomoto (Overland Park, KS, USA). L-isoleucine (98.5%) and L-valine (96.5%) were obtained from Ajinomoto Health & Nutrition North America, Inc. (Raleigh, NC, USA). Chromium oxide was purchased from Fisher Scientific (Bartlesville, OK, USA). ^6^ Mineral and vitamin premix was obtained from Nutra Blend, LLC (Neosho, MO, USA). The premix provided per kg of mix (MIN): manganese, 4.0%; zinc, 4.0%; iron, 2.0%; copper, 4500 ppm; iodine, 600 ppm; selenium, 60 ppm; vitamin A, 3,086,474.19 IU; vitamin D_3_, 1,102,317.07 ICU; vitamin E, 6613.9 IU; vitamin B12, 4.41 mg; menadione, 330.70 mg; riboflavin, 2645.55 mg; D-pantothenic acid, 2645.54 mg; niacin, 11,023.12 mg; vitamin B6, 551.16 mg; folic acid, 275.58 mg; choline, 154,323.71 mg; biotin, 13.23 mg.

## Data Availability

Datasets supporting the results of this article are included within the article and its Supplementary Files.

## Supplementary Materials

The supporting information can be downloaded at: https://www.mdpi.com/article/10.3390/ijms25084369/s1.
