# Supplementary material for: Low-Protein Diets Differentially Regulate Energy Balance during Thermoneutral and Heat Stress in Cobb Broiler Chicken (Gallus domesticus)
Source: Int J Mol Sci. 2024 Apr 15;25(8):4369. doi: 10.3390/ijms25084369 (PMC11050574; doi:10.3390/ijms25084369)
Supplement: Supplementary file 1 [file ijms-25-04369-s001.zip › ijms-2902129-supplementary.pdf]

Supplementary Materials

Supplementary Table S1. Phase growth performance of broilers fed with low protein diets during experimentally induced heat stress

Items	Treatments[1]				SEM[2]	*p*-Values		
	NPTN[2]	LPTN[2]	NPHS[2]	LPHS[2]		Diet	Temp[2]	Diet × Temp
ADG[3], g/d								
Grower	68.0	67.3	53.3	36.9*	1.8	≤ 0.01	≤ 0.01	≤ 0.01
Finisher	84.5	78.7	50.8	44.7#	3.7	0.03	≤ 0.01	0.96
ADFI[3], g/d								
Grower	145.7	156.7	149.7	120.6*	4.4	0.25	0.05	≤ 0.01
Finisher	198.0	202.8	160.7	144.8	5.5	0.46	≤ 0.01	0.18
ADPI[3], g/d								
Grower	28.3	22.1*	29.0	17.0*	1.0	≤ 0.01	0.09	0.02
Finisher	34.1	25.8*	27.6	18.4*	1.1	≤ 0.01	≤ 0.01	0.68
ADWI[3], mL/d								
Grower	256.0	203.8*	272.8	238.4*	4.8	≤ 0.01	≤ 0.01	0.03
Finisher	382.8	278.2*	408.2	319.2*	9.6	≤ 0.01	≤ 0.01	0.33
G:F[3], g/g								
Grower	0.4	0.4	0.4	0.4	0.01	0.28	0.03	0.79
Finisher	0.4	0.4#	0.3	0.3	0.01	0.04	≤ 0.01	0.19
G:P[3], g/g								
Grower	2.2	2.9*	2.0	2.5*	0.09	≤ 0.01	0.03	0.55
Finisher	2.6	3.2*	1.8	2.3*	0.10	≤ 0.01	≤ 0.01	0.84
W:F[3], mL/g								
Grower	1.8	1.4*	1.9	2.0	0.06	0.23	≤ 0.01	≤ 0.01
Finisher	2.0	1.4*	2.6	2.2*	0.09	≤ 0.01	≤ 0.01	0.19

[1] The values are the mean; *n*=9 pens (5-6 birds/pen).
[2] NPTN: normal protein diet under thermoneutral; LPTN: low protein diet under thermoneutral; NPHS: normal protein diet under heat stress; LPHS: low protein diet under heat stress; SEM: standard error of means; Temp: temperature.

[3] ADG: average daily gain; ADFI: average daily feed intake; ADPI: average daily protein intake; ADWI: average daily water intake; G:F: gain: feed; G:P: gain: protein; W:F: water: feed. The *p*-Values for the overall model effect for diet, temp, phase, diet × temp, diet × phase, temp × phase and diet × temp × phase for ADG were 0.59, 0.62, 0.05, 0.03, 0.58, 0.84, 0.17, for ADFI were 0.26, ≤ 0.01, ≤ 0.01, 0.02, 0.68, ≤ 0.01, 0.26, for ADPI were ≤ 0.01, ≤ 0.01, ≤ 0.01, 0.11, 0.79, ≤ 0.01, 0.05, for ADWI were ≤ 0.01, ≤ 0.01, ≤ 0.01, 0.11, ≤ 0.01, 0.31, 0.89, for G:F were 0.08, ≤ 0.01, ≤ 0.01, 0.71, 0.92, ≤ 0.01, 0.33 for G:P were ≤ 0.01, ≤ 0.01, 0.66, 0.57, 0.70, ≤ 0.01, 0.64 for W:F were ≤ 0.01, ≤ 0.01, ≤ 0.01, ≤ 0.01, ≤ 0.01, ≤ 0.01 and 0.06.

[*] Within rows, NPTN vs LPTN and NPHS vs LPHS $p \leq 0.05$

[#] Within rows, NPTN vs LPTN and NPHS vs LPHS $0.05 < p \leq 0.10$

Supplementary Table S2. Non-significant plasma metabolites in broilers fed with low protein diets during experimentally induced heat stress

Metabolites	Treatments[1]					*p*-Values		
	NPTN[2]	LPTN[2]	NPHS[2]	LPHS[2]	SEM[2]	Diet	Temp[2]	Diet × Temp[2]
Aconitic acid	10961	7194	10977	6944*	641	0.24	0.71	0.15
Adenosine	554	457	1513	749	175	0.38	0.06	0.19
Adenosine-5-monophosphate	1836	1506	1951	1420	158	0.81	0.77	0.24
Alloxanoic acid	1102	685	739	737	66	0.80	0.51	0.49
Aminovaleric acid	27753	10081	6239	9171*	4320	0.72	0.18	0.29
Anhydro-D-galactose	2743	1977	1864	2013	115	0.12	0.18	0.41
Arachidonic acid	8050	4361	4972	4339	416	0.32	0.07	0.19
Beta alanine	30291	24373	25862	22923	1952	0.59	0.73	0.60
Cellobiose	6968	4463	5279	4829	372	0.73	0.60	0.69
Chlorogenic acid	387	286	539	332	55	0.59	0.38	0.36
Cholesterone	929	489	589	533	50	0.54	0.32	0.32
Citric acid	1365683	953893	1149390	819214	68579	0.75	0.17	0.19
Cysteine glycine	2208	1692	2049	1597	80	0.57	0.88	0.23
Deoxy-5-methylthioadenosine	1019	674	959	883	53	0.67	0.25	0.93
Deoxypentitiol	4888	3695	4574	3885	206	0.45	0.60	0.57
Fructose	121412	19170	12677	18955	20703	0.29	0.17	0.20
Fucose	40990	24241	37045	28479	1662	0.17	0.18	0.95
Glucose	6775063	3551424	5890083	4807472	309293	0.22	0.32	0.39
Glutamic acid	661273	430980	798974	514513	46183	0.34	0.08	0.32
Glyceric acid	57758	49405*	55774	46473	2486	0.24	0.66	0.09
Glycolic acid	25366	19062	20280	16428	879	0.54	0.07	0.22
Guanosine	1433	774	989	1412	133	0.31	0.36	0.12
Hydroxybutanoic acid	171810	114226	149515	95181	12021	0.44	0.46	0.49
Hydroxycarbamate	3143	2602	2155	2434	161	0.07	0.17	0.97
Hydroxy-3-methylglutaric acid	1608	986	1040	993	74	0.82	0.08	0.30
Hydroxyphenylacetic acid	1652	1031	1011	982	81	0.74	0.06	0.47

Hydroxyphenyl propionic acid	3994	1855	1916	1291	362	0.25	0.06	0.58
Hydroxypropionic acid	41671	25135	30334	27577	1791	0.91	0.43	0.52
Indole-3-acetate	5263	8257*	4962	9756	1094	≤ 0.01	0.78	0.96
Indole-3-lactate	3608	3801	3209	2540	294	0.28	0.22	0.12
Isocitric acid	49960	29311	37732	29183	2566	0.31	0.24	0.89
Inosine-5-monophosphate	1964	883#	1721	1702	158	0.70	0.10	0.16
Lactic acid	4913087	3014734	4598820	3418810	322820	0.64	0.58	0.81
Maleic acid	1982	1379	2136	1345	110	0.45	0.36	0.21
Mannose	175608	84619	155980	117915	11786	0.28	0.34	0.54
Methyl-O-D-galactopyranoside	8834	5384	8560	7829	490	0.97	0.09	0.51
Myoinositol	435208	435208*	723105	608941	38954	0.20	0.22	0.17
N-acetylaspartic acid	7102	4839	6317	5656	448	0.78	0.63	1.00
N-acetylornithine	14447	9827	14836	14101	887	0.68	0.10	0.55
Nicotinic acid	1966	5647*	5577	5213	2336	0.23	0.10	0.26
Oxalic acid	103175	68793	64218	61229	6617	0.78	0.16	0.79
Pantothenic acid	5718	3054	4274	3734	333	0.56	0.96	0.32
Pentose	7030	5112	6071	5563	395	0.47	0.82	0.87
Phenylalanine	604111	375028	549289	392191	22022	0.14	0.68	0.50
Phenylethylamine	195180	129800	114912	147009*	11349	0.24	0.26	0.23
Phenyllactic acid	4071	1404	2752	1404*	354	0.19	0.09	0.54
Phosphate	2673412	1835797	1980977	1838620	80122	0.28	0.08	0.67
Phosphoglycerate	2218	1445	1229	1548	153	0.34	0.26	0.30
Picolinic acid	1964	1602#	1041	1805*	214	0.53	0.06	0.86
Putrescine	16802	8579	27303	19258	3801	0.56	0.12	0.88
Quinolinic acid	754	679	970	708	42	0.55	0.18	0.15
Shikimic acid	17727	7809*	17286	14991	1344	0.29	0.06	0.28
Thymine	1573	923	1052	1127	73	0.71	0.78	0.08
Xanthosine	805	518	1309	837	97	0.71	0.78	0.08
Xylonic acid	1559	918	1359	1013	84	0.24	0.72	0.81
Xylulose	19029	4141#	6905	5324	500	0.08	0.61	0.32

[1]The values are the mean peak height; *n*=8 per treatment

[2]NPTN: normal protein diet under thermoneutral; LPTN: low protein diet under thermoneutral; NPHS: normal protein diet under heat stress; LPHS: low protein diet under heat stress; SEM: standard error of means; Temp: temperature.

*Within rows, NPTN vs LPTN and NPHS vs LPHS $p \leq 0.05$

Within rows, NPTN vs LPTN and NPHS vs LPHS $0.05 < p \leq 0.10$

Supplementary Table S3. Quantitative PCR (qPCR) primer sequences, location on template, length, and GenBank accession number used in this study

Genes[1]	Sequence (5' → 3')	GenBank accession No.	Location on template (bp)	Amplicon length
Ghrelin	F-CCTTGGGACAGAAACTGCTC<br>R- CACCAATTTCAAAAGGAACG	NM_001001131.1	199-218<br>382-401	203
CCK	F-CAGCAGAGCCTGACAGAACC<br>R-AGAGAACCTCCCAGTGGAACC	NM_001001741.1	162-181<br>309-329	168
PYY	F-AGGAGATCGCGCAGTACTTCTC<br>R-TGCTGCGCTTCCCATACC	NM_001361182.1	144-166<br>205-222	78
Secretin	F-TGAGTTGGCTGAGAGTACAG<br>R-CTTCACATCTGTCACCAGCT	NM_001024833.2	3-22<br>570-589	587
GIP	F-CGCAGTGAGTGACCAAAGC<br>R-TAGGAGCCATGCAAGGAAGT	NM_001080104.1	366-384<br>413-432	67
β1-AR	F-CTGGCACCTAGCACAATGAA<br>R-CTGCTTGCTGATCCACATCT	NM_205518.1	1026-1045<br>1129-1148	123
PGC-1α	F-GGGACCGGTTTGAAGTTTTTG<br>R-GGCTCGTTTGACCTGCGTAA	NM_001006457.1	2072-2092<br>2203-2222	151
AMPKα1	F-ATCTGTCTCGCCCTCATCCT<br>R-CCACTTCGCTCTTCTTACACCTT	NM_001039603.1	1337-1356<br>1439-1461	125
β -Actin	F- CAATGGCTCCGGTATGTGCA<br>R- AGGCATACAGGGACAGCACA	NM_205518.1	101-120<br>482-501	401
Sirtuin 1	F-<br>GATCAGCAAAAGGCTGGATGGT<br>R-ACGAGCCGCTTTCGCTACTAC	NM_001004767.1	1932-1953<br>2054-2074	143
Cox IV	F-CTTTCCACCTCCATCTGTGTGA<br>R-TGCTGGATGGCTGAAATCG	NM_001030577.1	83-104<br>139-157	75

[1]CCK: cholecystokinin, PYY: peptide YY, GIP: gastric inhibitory polypeptide, β1-AR: β1-adrenergic receptor, PGC-1α: peroxisome proliferator-activated receptor-gamma coactivator, AMPKα1: AMP-activated protein kinase α1, β-Actin: beta actin, Cox IV: cytochrome c oxidase subunit IV

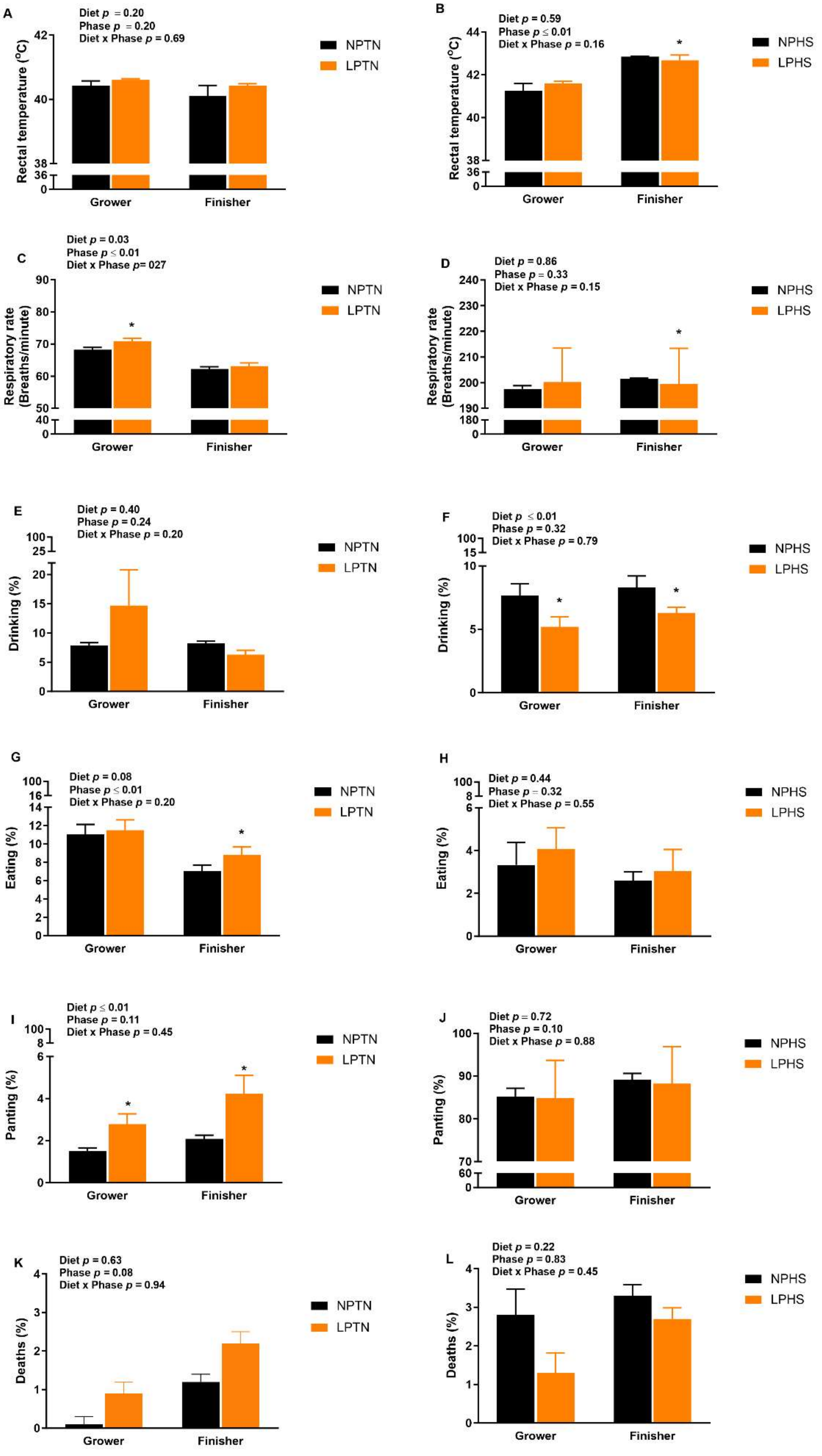
A
Diet p = 0.20
Phase p = 0.20
Diet x Phase p = 0.69
Rectal temperature (ºC)
NPTN
LPTN
Grower
Finisher
B
Diet p = 0.59
Phase p ≤ 0.01
Diet x Phase p = 0.16
Rectal temperature (ºC)
NPHS
LPHS
Grower
Finisher
C
Diet p = 0.03
Phase p ≤ 0.01
Diet x Phase p= 027
Respiratory rate (Breaths/minute)
NPTN
LPTN
Grower
Finisher
D
Diet p = 0.86
Phase p = 0.33
Diet x Phase p = 0.15
Respiratory rate (Breaths/minute)
NPHS
LPHS
Grower
Finisher
E
Diet p = 0.40
Phase p = 0.24
Diet x Phase p = 0.20
Drinking (%)
NPTN
LPTN
Grower
Finisher
F
Diet p ≤ 0.01
Phase p = 0.32
Diet x Phase p = 0.79
Drinking (%)
NPHS
LPHS
Grower
Finisher
G
Diet p = 0.08
Phase p ≤ 0.01
Diet x Phase p = 0.20
Eating (%)
NPTN
LPTN
Grower
Finisher
H
Diet p = 0.44
Phase p = 0.32
Diet x Phase p = 0.55
Eating (%)
NPHS
LPHS
Grower
Finisher
I
Diet p ≤ 0.01
Phase p = 0.11
Diet x Phase p = 0.45
Panting (%)
NPTN
LPTN
Grower
Finisher
J
Diet p = 0.72
Phase p = 0.10
Diet x Phase p = 0.88
Panting (%)
NPHS
LPHS
Grower
Finisher
K
Diet p = 0.63
Phase p = 0.08
Diet x Phase p = 0.94
Deaths (%)
NPTN
LPTN
Grower
Finisher
L
Diet p = 0.22
Phase p = 0.83
Diet x Phase p = 0.45
Deaths (%)
NPHS
LPHS
Grower
Finisher

Supplementary Figure S1. Behavioral adaptations of broilers fed with low protein diets during experimentally induced heat stress

Effect of low protein diets on rectal temperature (A and B), respiratory rate (C and D), percent of birds drinking (E and F), percent of birds eating (G and H), percent of birds panting (I and J) and percent death (K and L) in broilers during thermoneutral (TN) (A, C, E, G, I and K) and heat stress (HS) (B, D, F, H, J and L). NPTN: normal protein diet under thermoneutral; LPTN: low protein diet under thermoneutral; NPHS: normal protein diet under heat stress; LPHS: low protein diet under heat stress. The *p*-Values for the overall model effects of diet, temp, time, diet × temp, diet × time, time × temp, diet × temp × time for rectal temperature were 0.19, ≤ 0.01, ≤ 0.01, 0.55, 0.47, ≤ 0.01, 0.20, for respiratory rate were 0.34, ≤ 0.01, ≤ 0.01, 0.52, 0.07, ≤ 0.01 and 0.44, for percent of birds drinking were 0.93, 0.09, 0.33, 0.10, 0.20, 0.14, and 0.16, for percent of birds eating were 0.13, ≤ 0.01, ≤ 0.01, 0.64, 0.71, 0.08 and 0.56, for percent of birds panting were 0.61, ≤ 0.01, 0.05, 0.24, 0.95, 0.26 and 0.74 and percent death were 0.47, 0.56, 0.66, 0.22, 0.53, 0.94, and 0.50. The values are the mean ± SEM; *n*=9 pens (5-6 birds/pen).

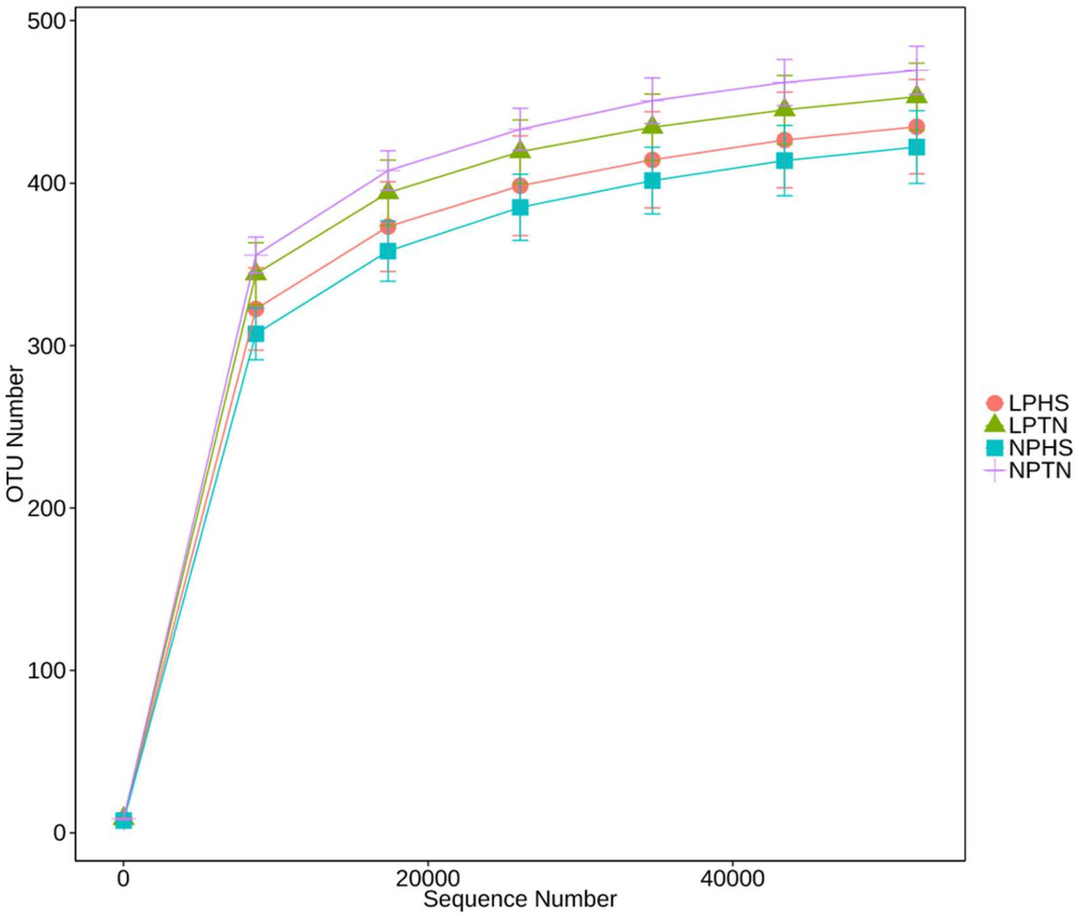

Supplementary Figure S2. Cecal rarefaction curve analysis for broilers fed with low protein diets during experimentally induced heat stress

The rarefaction curves from cecal samples represent the number of operational taxonomic units (OTU) as a function of the number of reads sampled. NPTN: normal protein diet under thermoneutral; LPTN: low protein diet under thermoneutral; NPHS: normal protein diet under heat stress; LPHS: low protein diet under heat stress. *n*=6 for each treatment.

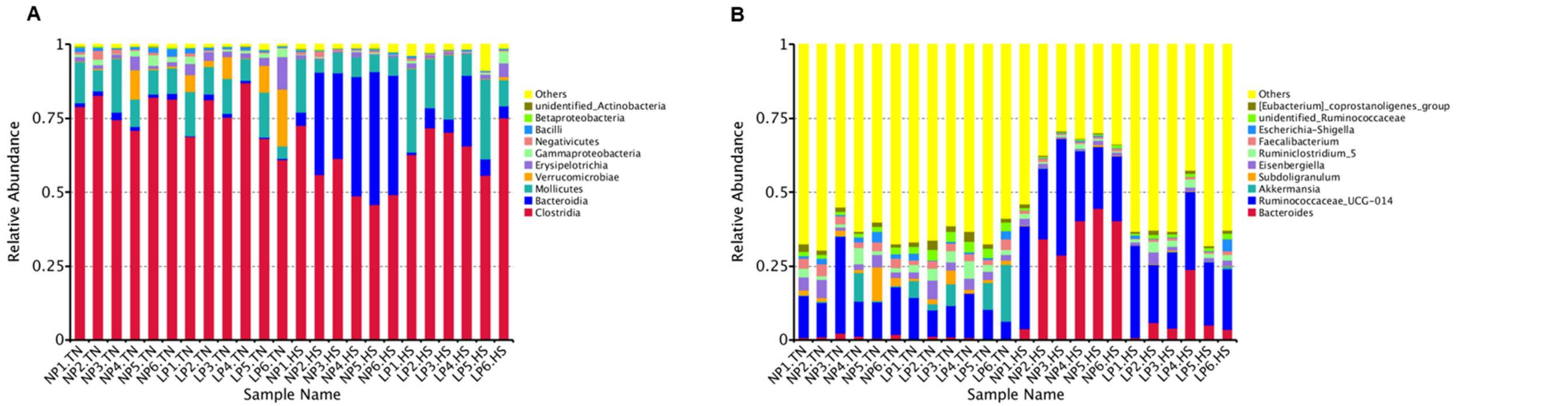

Supplementary Figure S3. The composition of cecal bacterial populations in broilers fed with low protein diets during experimentally induced heat stress

The relative abundance of bacterial community composition in cecal samples of individual pigs at phylum level (A) and at genus level (B). NPTN: normal protein diet under thermoneutral; LPTN: low protein diet under thermoneutral; NPHS: normal protein diet under heat stress; LPHS: low protein diet under heat stress. For clarity reasons, only the top 10 phyla and genera are shown. *n*=6 for each treatment.

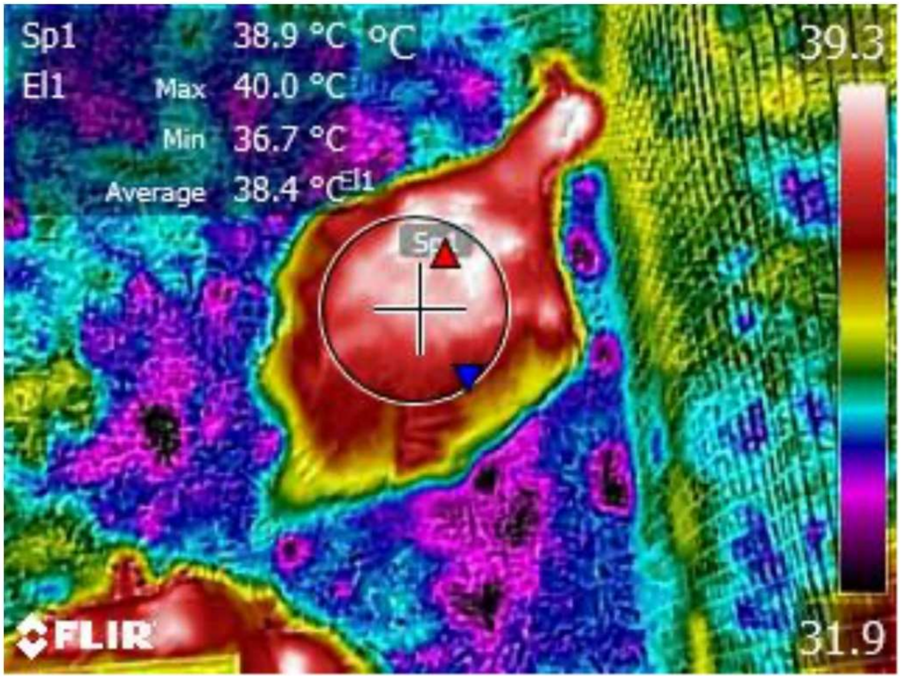

Supplementary Figure S4. A representative screenshot of a thermal image from FLIR Research Studio.

The region of interest was determined by drawing a circle in the back of birds. The mean temperature data from the region of interest were extracted from birds fed with normal protein and low protein diets at different time points during thermoneutral and heat stress periods and used for data analysis. FLIR Research Studio (version 5.13.18031.2002).
